# The Impact of Dietary Interventions on the Microbiota in Inflammatory Bowel Disease: A Systematic Review

**DOI:** 10.1093/ecco-jcc/jjad204

**Published:** 2023-12-15

**Authors:** Cheenie Nieva, Jennifer Pryor, Georgina M Williams, Emily C Hoedt, Grace L Burns, Guy D Eslick, Nicholas J Talley, Kerith Duncanson, Simon Keely

**Affiliations:** College of Health, Medicine and Wellbeing, University of Newcastle, Callaghan, NSW, Australia; National Health and Medical Research Council [NHMRC], Centre of Research Excellence in Digestive Health, University of Newcastle, Newcastle, NSW, Australia; Immune Health Research Program, Hunter Medical Research Institute, New Lambton Heights, NSW, Australia; College of Health, Medicine and Wellbeing, University of Newcastle, Callaghan, NSW, Australia; National Health and Medical Research Council [NHMRC], Centre of Research Excellence in Digestive Health, University of Newcastle, Newcastle, NSW, Australia; Immune Health Research Program, Hunter Medical Research Institute, New Lambton Heights, NSW, Australia; College of Health, Medicine and Wellbeing, University of Newcastle, Callaghan, NSW, Australia; National Health and Medical Research Council [NHMRC], Centre of Research Excellence in Digestive Health, University of Newcastle, Newcastle, NSW, Australia; Immune Health Research Program, Hunter Medical Research Institute, New Lambton Heights, NSW, Australia; College of Health, Medicine and Wellbeing, University of Newcastle, Callaghan, NSW, Australia; National Health and Medical Research Council [NHMRC], Centre of Research Excellence in Digestive Health, University of Newcastle, Newcastle, NSW, Australia; Immune Health Research Program, Hunter Medical Research Institute, New Lambton Heights, NSW, Australia; College of Health, Medicine and Wellbeing, University of Newcastle, Callaghan, NSW, Australia; National Health and Medical Research Council [NHMRC], Centre of Research Excellence in Digestive Health, University of Newcastle, Newcastle, NSW, Australia; Immune Health Research Program, Hunter Medical Research Institute, New Lambton Heights, NSW, Australia; College of Health, Medicine and Wellbeing, University of Newcastle, Callaghan, NSW, Australia; National Health and Medical Research Council [NHMRC], Centre of Research Excellence in Digestive Health, University of Newcastle, Newcastle, NSW, Australia; College of Health, Medicine and Wellbeing, University of Newcastle, Callaghan, NSW, Australia; National Health and Medical Research Council [NHMRC], Centre of Research Excellence in Digestive Health, University of Newcastle, Newcastle, NSW, Australia; Immune Health Research Program, Hunter Medical Research Institute, New Lambton Heights, NSW, Australia; College of Health, Medicine and Wellbeing, University of Newcastle, Callaghan, NSW, Australia; National Health and Medical Research Council [NHMRC], Centre of Research Excellence in Digestive Health, University of Newcastle, Newcastle, NSW, Australia; Immune Health Research Program, Hunter Medical Research Institute, New Lambton Heights, NSW, Australia; College of Health, Medicine and Wellbeing, University of Newcastle, Callaghan, NSW, Australia; National Health and Medical Research Council [NHMRC], Centre of Research Excellence in Digestive Health, University of Newcastle, Newcastle, NSW, Australia; Immune Health Research Program, Hunter Medical Research Institute, New Lambton Heights, NSW, Australia

**Keywords:** Inflammatory bowel disease, diet, microbiome

## Abstract

**Background and Aims:**

Diet plays an integral role in the modulation of the intestinal environment, with the potential to be modified for management of individuals with inflammatory bowel disease [IBD]. It has been hypothesised that poor ‘Western-style’ dietary patterns select for a microbiota that drives IBD inflammation and, that through dietary intervention, a healthy microbiota may be restored. This study aimed to systematically review the literature and assess current available evidence regarding the influence of diet on the intestinal microbiota composition in IBD patients, and how this may affect disease activity.

**Methods:**

MEDLINE, EMBASE, Scopus, Web of Science, and Cochrane Library were searched from January 2013 to June 2023, to identify studies investigating diet and microbiota in IBD.

**Results:**

Thirteen primary studies met the inclusion criteria and were selected for narrative synthesis. Reported associations between diet and microbiota in IBD were conflicting due to the considerable degree of heterogeneity between studies. Nine intervention studies trialled specific diets and did not demonstrate significant shifts in the diversity and abundance of intestinal microbial communities or improvement in disease outcomes. The remaining four cross-sectional studies did not find a specific microbial signature associated with habitual dietary patterns in IBD patients.

**Conclusions:**

Diet modulates the gut microbiota, and this may have implications for IBD; however, the body of evidence does not currently support clear dietary patterns or food constituents that are associated with a specific microbiota profile or disease marker in IBD patients. Further research is required with a focus on robust and consistent methodology to achieve improved identification of associations.

## 1. Introduction

Inflammatory bowel diseases [IBD] encompass a collection of chronic, relapsing-remitting disorders of the gastrointestinal [GI] tract, characterised by an inflammatory process that requires extensive medical and surgical management. Crohn’s disease [CD] and ulcerative colitis [UC] are the two most prevalent manifestations of IBD and affect up to 0.5% of the global population.^1,2^ Although the aetiology of IBD is yet to be fully elucidated, the current pathogenesis paradigm involves a complex interplay between multiple factors, including genetic susceptibility and environmental triggers.^3^ Among these environmental exposures, diet may be a key driver of intestinal inflammation, as it plays a fundamental role in modulating the intestinal milieu and physiology, with modifications to diet strongly influencing the intestinal microbiota composition and function.^4,5^ Excessive changes to the intestinal microbiota may result in a disrupted equilibrium between commensal and harmful microbes, often referred to as ‘dysbiosis’ and characterised by a reduction in microbial diversity.^6^ Microbial dysbiosis has been shown to perturb intestinal barrier integrity, which facilitates the translocation of potentially antigenic microbiota and diet-derived components into the underlying mucosa, thus driving an aberrant immune response and an ongoing inflammatory cycle.^7^

IBD patients often exhibit intestinal microbiota composition which significantly differs to that of healthy individuals.^8–11^ Although a specific pattern of dysbiosis in IBD patients has not been established, numerous studies have reported a decrease in the abundances of bacterial taxa within the phyla Firmicutes^8,12,13^ and Bacteroides,^9,14^ and an increase in Proteobacteria.^15–17^ Furthermore, diet may also exert functional changes to the microbiota by facilitating the production of metabolites such as short chain fatty acids [SCFAs] which are known to have an immune regulatory role and serve as a primary energy source for intestinal epithelial cells.^18^ This underscores diet as a key modifiable factor that can directly influence the GI environment.

The efficacy of exclusive enteral nutrition [EEN] [which replaces solid foods with a nutritionally complete liquid formula for up to 8 weeks] in paediatric patients with active CD^19^ provides the strongest evidence for the link between dietary exposure and disease.^20,21^ However, EEN is underused in adult populations^22^ due to unpalatability, low tolerability and poor compliance with such a restrictive diet.^22,23^ Therefore, dietary approaches that allow access to whole foods, to maximise food variety, while optimally mitigating symptoms need to be explored. To date, evidence regarding the implementation of predefined diets [ie, dietary regimens that involve a significant restriction or complete exclusion of one or more suspect food groups] for ameliorating the course of IBD is conflicting and inconsistent.^24,25^ Many patients perceive diet to be an initiating factor in their disease or in evoking relapse, and have long sought dietary advice as part of IBD management.^26^ However, there is still no consensus nor clear indications toward an optimal dietary therapy beyond healthy eating guidelines, due to limited available research data.^27^

The Western-style dietary pattern is considered a predominant trigger implicated in the development of IBD, however, the specific dietary components responsible are yet to be identified.^28,29^ This dietary pattern, which is typically devoid in fibre from fruits and vegetables and rich in saturated fat and digestible carbohydrates, tends to evoke dysbiosis [reduced microbial diversity], resulting in dysregulated production of SCFAs by bacteria in the gut, which has profound effects on maintaining intestinal homeostasis.^30,31^ Additionally, this diet facilitates the expansion and activity of colonic mucus-degrading bacteria, which drives intestinal inflammation and gut barrier impairment.^30,32^ Dietary therapy has the potential to manipulate the gut microbiota to restore a healthy profile and alleviate intestinal inflammation in IBD patients. Therefore, the impact of diet on the intestinal microbiota and its potential to shift the future course of the disease should be considered when developing nutritional guidelines for IBD patients. An updated 2022 systematic review and meta-analysis analysed 27 controlled trials of solid food diets for the induction or maintenance of remission in IBD, and concluded that currently available evidence remains low.^33^ Moreover, this study did not consider the potential impact of modulation of the intestinal microbiota, through diet, on disease outcomes. Given this knowledge gap, the aim of this review is to describe relationships between whole-food dietary exposure and the intestinal microbiota in adult patients with IBD, and whether this influences disease activity.

## 2. Methods

A systematic literature review was conducted in agreement with the Preferred Reporting Items for Systematic reviews and Meta-Analyses [PRISMA] guidelines^34^ and by using the PRISMA 2020 Checklist [Supplementary Table S1]. Details of the methods, including the search strategy, eligibility criteria, extraction process, and analysis, were registered with the International Prospective Register of Systematic Reviews [registration no. CRD42022292986; http://www.crd.york.ac.uk/PROSPERO].^35^ The PICOS [Participants, Interventions, Comparisons, Outcomes and Study design] model was used to define the research question [Supplementary Table S2].

### 2.1. Search strategy

A systematic literature search was performed in the electronic databases MEDLINE, EMBASE, Cochrane Library, Scopus, and Web of Science, using a combination of free-text words, medical subject headings [MeSH] terms, and synonyms relevant to this review, with the assistance of two experienced research librarians [AS and JB]. Our search strategy included terms related to [i] inflammatory bowel diseases, [ii] microbiome/microbiota, and [iii] diet/food/nutrition [Supplementary Table S3]. To ensure the studies reflected contemporary microbiome sequencing techniques, the search strategy was restricted to those published after the completion of the first phase of the Human Microbiome Project in June 2012, which characterised the microbial communities from five major sites of the human body including the GI tract.^36,37^ The databases were searched from January 1, 201 to June 6, 2023, and limited to the English language, as translation services were not available. Abstracts, review articles, editorials, case reports, case series, theses, and study protocols were excluded.

### 2.2. Study selection

Search results were collated and merged into the reference management software EndNote 20 [Thompson Reuters], and de-duplicated before uploading to Covidence systematic review software [Veritas Health Innovation, Melbourne, Australia; https://www.covidence.org]. Title and abstract screening were conducted by two reviewers [CN and JP or KD], with conflicts resolved by third reviewer [JP or KD]. Full-text articles were retrieved and uploaded into Covidence for full-text review by two reviewers [CN and KD], with conflicts resolved by third reviewer [JP or ECH]. Studies were included if they complied with each of the following inclusion criteria: [i] inclusion of adult patients [≥18 years of age] with IBD of any type; [ii] study of the effect of diet [either using a dietary intervention or examining habitual diet] on the composition of the intestinal microbiota; and [iii] measurement of microbiota composition by 16S or shotgun metagenomic sequencing. Studies solely investigating the following were excluded: [i] paediatric populations [≤18 years of age]; [ii] enteral nutrition; [iii] animal studies; [iv] use of other microbiota sequencing techniques.

### 2.3. Data extraction

Relevant data were manually extracted from eligible studies by one author [CN] into tabular form. Data extracted included: demographic characteristics of the participants [eg, age, sex, IBD type, and degree of disease activity]; outcomes specific to each study [eg, changes in GI symptoms, clinical disease activity, quality of life, and inflammatory markers]; study design [eg, location, type of design, number of participants]; characteristics of diet [eg, type of dietary intervention or habitual diet intake, comparator, study duration, details of baseline, washout and end of study periods, dietary assessment methods, outcomes]; microbiota composition [eg, specimen type, sample site, DNA extraction method, sequencing techniques, changes in alpha and beta diversity and relative microbial abundances] [Supplementary Table S2]. Data extraction was checked by a second reviewer [KD or GDE or ECH or GMW].

### 2.4. Risk of bias assessment

Quality appraisal of all included studies was undertaken to reduce the risk of bias [Supplementary Table S4]. Risk of bias was independently assessed during the selection of studies by two reviewers [CN and JP] using the Joanna Brigg’s Institute [JBI] critical appraisal tools for randomised controlled trials [RCTs] [13 criteria], and quasi-experimental [nine criteria] and cross-sectional studies [eight criteria].^38^ JBI includes questions about suitability of the study sample and selection, description of subjects and setting, completeness of presented data and analysis, appropriateness of measuring the condition, and identification of confounding factors.

### 2.5. Data availability statement

The data underlying this article are available in the article and in its online Supplementary material.

## 3. Results

**Figure 1:**
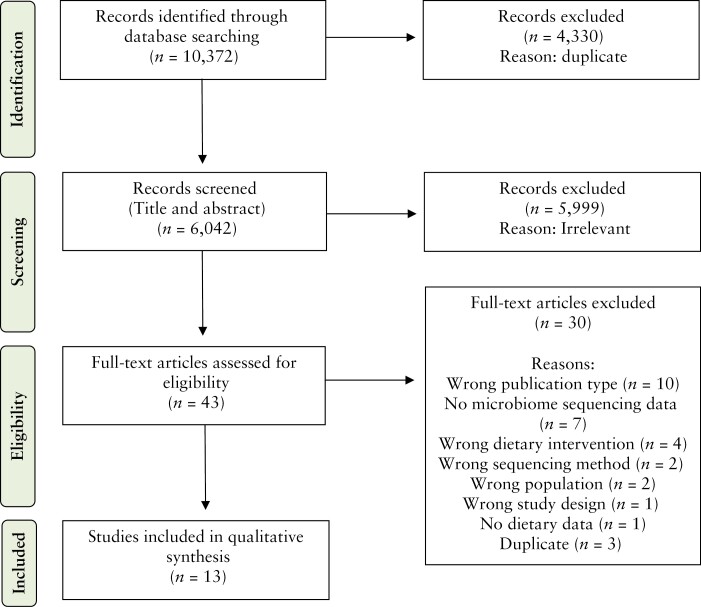
Flow diagram depicting records identified, included, and excluded for review and the reasons for exclusion.

The systematic search identified 10 372 records, of which 4330 were removed due to duplication. The remaining 6042 records were screened on title and abstract and 5999 were excluded. The remaining 43 studies were assigned to full-text screening, of which 13^39–51^ were found to comply with the eligibility criteria and were included in the review [Figure 1].

### 3.1. Risk of bias

Overall, 100% [*n *= 9] of the intervention studies [*n *= 7 RCTs and *n *= 2 quasi-experimental]^39–44,49–51^ met at least 60% [8/13 and 7/9, respectively] of the JBI study quality criteria [Supplementary Table S4]. RCTs scored poorly for criteria relating to blinding of participants and evaluators to allocation and/or treatment, due to the nature of food-based dietary interventions. Of cross-sectional studies,^45,47,48^ 75% [*n *= 3 of 4] satisfied at least five of eight items, with one study scoring as low as two of eight. For 50% [*n *= 2 of 4]^46,48^ of these, potential biases stemmed from the lack of validated or reliable measures and neglecting to identify and adjust for confounding factors. According to these assessments, the overall methodological quality of included studies was deemed to be moderate.

### 3.2. General study characteristics

Geographically, six of the 13 studies included patients from North America,^40,41,44,49–51^ five from Europe,^39,43,45–47^ and three from Asia^42,43,48^ [Table 1]. The publication dates ranged from 2019 to 2023. Of the included studies, nine were experimental, of which six were RCTs,^39–43,49,50^ and two were non-randomised interventions.^44,51^ The remaining four studies were of cross-sectional design. Of the seven randomised trials, two were single-blinded, one adopted a parallel cross-over design, and two were open-label trials.

**Table 1:** Characteristics of randomised trials and cross-sectional studies for inflammatory bowel diseases

First author	Country	Year	Study design	Study intervention group	Sample size	Mean age, y	Sex, male, *n* [%]	Disease state	Study comparator group	Sample size	Mean age, y	Sex, male, *n* [%]	Primary outcome/aim	Duration/ recruitment period
Cox^39^	UK	2020	Randomised, placebo-controlled, single-blind, trial	Low FODMAP	CD, *n* = 14 UC, *n* = 13	33	10 [37]	Remission	Sham diet	CD, *n* = 12 UC, *n* = 13	40	13 [52]	Change in symptoms as measured by IBS-SSS	4 weeks
Fritsch^40^	USA	2021	Randomised, parallel group, cross-over trial	Low fat diet	UC, *n* = 18	42 [33–47]	7 [39]	Remission or mild to moderate	Improved standard American diet	UC, *n* = 18	42 [33–47]	7 [39]	Quality of life as measured by SIBDQ	8 weeks
Lewis^41^	USA	2021	Randomised, controlled, parallel group, trial	Mediterranean diet	CD, *n* = 92	37 [29–53]	41 [41]	Mild to moderate	Specific carbohydrate diet	CD, *n* = 99	36 [27–46]	29 [31]	Symptomatic remission at week 6 defined as sCDAI <150 in the absence of initiation or increase of any medications	12 weeks
Haskey^49^	Canada	2023	Randomised, controlled trial	Mediterranean diet	UC, *n* = 15	52	6 [40]	Remission	Canadian habitual diet	UC, *n* = 13	39	4 [31]	Reduction of the SCCAI >1.5 score by week 12	12 weeks
Strauss^50^	Canada	2023	Randomised controlled, open-label trial	Mediterranean diet	UC, *n* = 22	37 [21–80]	10 [45]	Remission and active	Habitual diet	UC, *n* = 18	37 [21–80]	11 [61]	Identify features of the intestinal microbiota associated with Mediterranean diet and changes in FC	8 weeks
Shabat^43^	Italy and Israel	2021	Randomised, controlled, single-blinded trial	Ulcerative colitis exclusion diet	UC, *n* = 15	33	11 [73]	Active moderate	UCED plus FT Free diet plus FT	UC, *n* = 17 UC, *n* = 19	44 43	14 [74] 12 [60]	Week 8 clinical steroid-free remission defined as SCCAI <3	12 weeks
Sahu^42^	India	2021	Randomised, controlled, open-label clinical trial	EEN	UC, *n* = 32	33	12 [37]	Acute severe	Standard of care [normal diet]	UC, *n* = 30	38	13 [43]	Corticosteroid failure defined by the need for salvage medical therapy or colectomy	7 days
Zhang^44^	Canada	2020	Controlled-intervention study	Mediterranean diet	CD, *n* = 26	48	9 [60]	Remission	Diversified diet [Mediterranean diet]	CD, *n* = 32	51	10 [40]	Characterise differences in microbiota composition and function	12 weeks
Olendzki^51^	USA	2022	Single-arm, pre-post intervention trial, prospective	IBD-AID	CD, *n* = 12 UC, *n *= 7	42 38	4 [27]	Mild, moderate, and active	Habitual diet	CD, *n* = 12 UC, *n *= 7	42 38	4 [27]	Establish whether IBD-AID can revert dysbiosis by favouring SCFA-producing bacterial species that are commonly depleted in IBD patients	8 weeks
Schreiner^46^^a^	Switzerland	2019	Cross-sectional, prospective	Vegetarian diet/ gluten-free diet	CD, *n* = 33 UC, *n* = 19/ CD, *n* = 31 UC, *n* = 26	Not reported/ Not reported	14 [30]/ 21 [37]	Remission and active	Non-vegetarian/ Non-gluten-free	CD, *n* = 660 UC, *n* = 542/ CD, *n* = 642 UC, *n* = 524	N/R N/R	582 [49] 560 [48]	Characterise differences in mucosa-associated microbiota composition based on dietary habits	2006–2015
Weng^48^	China	2019	Cross-sectional	Habitual diet	CD, *n* = 173 UC, *n* = 107	29 [21–39] 42 [30–53]	115 [67] 69 [65]	Not reported	Habitual diet	HC, *n* = 42	48 [38–52]	28 [67]	Investigate the impact of dietary preferences on intestinal microbiota composition	Apr 2014 – Apr 2015
Teofani^47^	Italy	2022	Cross-sectional	Habitual diet	CD, *n* = 52 UC, *n* = 58	42 43	28 [53] 29 [50]	Remission, mild and moderate	Habitual diet	HC, *n* = 42	38	15 [36]	Investigate the role of covariates and confounders such as dietary habits in modulating the intestinal microbiota composition	Apr 2019 – Feb 2020
Berbisá^45^	Faroe Islands	2022	Cross-sectional, matched	Habitual diet	UC, *n* = 41	48	18 [44]	Remission and active	Habitual diet	HC, *n* = 144	55	57 [40]	Characterise bacterial composition of intestinal microbiota	Oct 2018 – Jun 2019

y, years; FODMAP, fermentable oligosaccharides, disaccharides, monosaccharides, and polyols; CD, Crohn’s disease; UC, ulcerative colitis; IBS-SSS, Irritable Bowel Syndrome Severity System Scores; SIBDQ, Short Inflammatory Bowel Disease Questionnaire; sCDAI, short Crohn’s Disease Activity Index; MDSS, Mediterranean Diet Serving Score; UCED, Ulcerative Colitis Exclusion Diet; FT, faecal transplant; SCCAI, Simple Clinical Colitis Activity Index; EEN, exclusive enteral nutrition; FC, faecal calprotectin; IBD-AID, Inflammatory Bowel Disease-Anti-Inflammatory Diet; IBD, inflammatory bowel disease; HC, healthy control.

^a^For this study by Schreiner *et al*., the groups were taken from the same total cohort of 1313 individuals with IBD.

Five of the nine experimental studies investigated UC, two studies referred to CD, and two examined both disease types [Table 1]. Three of the four observational studies reported on both disease types and healthy controls [HC], and one was performed in a UC cohort only. The population analysed in the research studies included a total of 3427 individuals with IBD: 1878 diagnosed with CD and 1549 with UC, with sample sizes ranging between 15 and 1202 participants. All studies included both male and female participants, with the mean ages ranging from 29 to 53 years. Healthy mixed-sex cohorts were included in three of the four cross-sectional studies [total *n* = 228], with participant mean ages ranging from 48 to 55 years.

### 3.3. Cross-sectional studies’ characteristics and outcomes

The four cross-sectional studies investigated habitual dietary habits of IBD patients and healthy individuals [Table 2]. Three of the four studies evaluated the normal or habitual diet of IBD patients compared with healthy participants. The dietary assessment methods used were unique between studies and the food data analysis tools used were not reported in any of the studies except for Weng *et al.*^48^ This study demonstrated significantly lower intake of vegetables, fish, and shellfish, and higher intake of eggs, milk, and dairy products, in a cohort of IBD patients compared with HC, especially in CD patients. Comparing with HC, Teofani *et al*.^47^ also reported significantly lower intake of vegetables, legumes, fruits, and cereals, and high intakes of dairy products in IBD patients. Conversely, Berbisá *et al*.^45^ did not find any significant differences in the patterns of eating habits between UC patients and HC, with both groups consuming a Western-style dietary pattern. Schreiner *et al.*^46^ investigated restrictive dietary habits of IBD patients, and defined the participants as either being vegetarian, non-vegetarian [meat-eating], gluten-free diet, or non-gluten-free, based on the frequency of their meat or gluten consumption, respectively. However, this study did not further analyse differences in intakes of specific dietary components between groups.

**Table 2:** Summary of findings: dietary characteristics, and outcomes of cross-sectional studies.

First author, year	Diet type/ample size	Dietary assessment methosd	Dietary data time point	Food data analysis	Dietary outcomes [CD, UC, or HC]
Schreiner 2019^46^^a^	Vegetarian diet CD, *n* = 33 UC, *n* = 19	Self-report questionnaire	Annual follow-up	Not applicable	Not assessed
	Gluten-free diet CD, *n* = 31 UC, *n* = 26	As above	As above	Not applicable	Not assessed
	Non-vegetarian CD, *n* = 660 UC, *n* = 542	As above	As above	Not applicable	Not assessed
	Non-gluten free diet CD, *n* = 642 UC, *n* = 524	As above	As above	Not applicable	Not assessed
Weng 2020^48^	Habitual diet CD, *n* = 173 UC, *n* = 107	CNHS2010-F	1 year prior	1. China Nutrition Society nutrient tables 2. National Institute of Nutrition and Food Safety food nutrition calculator	↓ daily intake of vegetables^*^ ↓ fish and shellfish^*^ ↑ intake of eggs^*^ ↑ milk and dairy products [yoghurt]^*^ compared with HC, especially in CD
	Habitual diet HC, *n* = 42	As above	As above	As above	As above
Teofani 2022^47^	Habitual diet CD, *n* = 52 UC, *n* = 58	Questionnaire modified by an anamnestic interview according to the Italian diet and lifestyle habits	Not reported	Not applicable	↓ fruits, vegetables, and legumes^*^ ↓ dairy products^*^ ↓ cereals^*^ compared with HC, especially in CD
	Habitual diet HC, *n* = 42	As above			As above
Berbisá 2022	Habitual diet [Faroese diet] UC, *n* = 41	FFQ	3 months prior	Not applicable	Resembled a Western diet pattern. ↑ dairy starch, meat, vegetables, fruits, coffee, or tea, sweets, processed meats [cold meat cuts] ↓ Traditional Faroese foods [eg, fermented lamb, dried fish, pilot whale meat, and blubber]
	Habitual diet [Faroese diet] HC, *n* = 144	As above	As above	Not applicable	No differences in the pattern of eating habits as above

CD, Crohn’s disease; UC, ulcerative colitis; HC, healthy controls; CNHS2010-F, 2010 Chinese Residents of Nutrition and Health Status Monitoring Semi-Quantitative Food Questionnaire; FFQ, Food Frequency Questionnaire.

^a^For this study, Schreiner *et al*., the questionnaire assessed dietary habits, not intake.

^*^Indicates statistically significant *p*-values [*p* <0.05].

### 3.4. Dietary interventions’ characteristics and outcomes

A total of nine unique dietary patterns was identified across the nine diet intervention studies [Table 3]. These included a Mediterranean diet [MD], low FODMAP diet, sham [low FODMAP] diet, low fat diet [LFD], improved standard American diet [iSAD], specific carbohydrate diet [SCD], IBD-anti-inflammatory diet [IBD-AID],UC exclusion diet [UCED], and standard-of-care [SOC] diet. The majority of intervention studies had two trial arms,^39–42,44^ except for one single-arm study^51^ and one with three trial arms^43^ which included patients who underwent faecal transplant [FT] using microbiota obtained from healthy donors. The duration of the dietary interventions ranged from 7 days to 12 weeks. Disease states varied considerably within and between studies, from quiescent to active or mild to moderate or severe. Dietary data were captured at different time points and frequencies throughout the trials from baseline to end of trial. The dietary assessment and food analysis methods used to obtain information on dietary intake were highly variable. Three studies applied food diaries,^39,40,44^ four studies used diet adherence questionnaires,^41,43,49,50^ and five studies administered 24-h food recall questionnaires.^40,41,49–51^

**Table 3:** Summary of findings: dietary characteristics and outcomes of intervention studies.

First author, year	Diet type/sample size	Diet protocol inclusion	Diet protocol restrictions	Diet rationale	Dietary assessment method	Dietary data collection time point	Food data analysis	Dietary outcomes	
								Baseline versus end-of-trial [within group]	compared with other diet [between groups]
Cox 2020^39^	Low FODMAP diet CD, *n* = 14 UC, *n* = 13	Not applicable	Exclusion: fermentable oligosaccharides [fructans, galacto-oligosaccharides] disaccharides [lactose], monosaccharides [fructose] and polyols [sorbitol and mannitol]	Dietary restriction of easily fermentable but poorly absorbed carbohydrates may ameliorate functional gut symptoms by reducing diet-induced luminal water and colonic gas	7–day food diary	End of trial [Week 4]	1 Nutritics [nutrient and fibre intakes] [https://wwwnutriticscom/en/] [Nutritics, Dublin, Ireland] 2 Bespoke Database [assess FODMAP intake] [Monash University, Melbourne, Australia]	Compliance: 88%	↓ FODMAP [fructans, GOS, lactose, excess fructose, sorbitol, and mannitol] ↓ protein, fat and sugar intake, and energy compared with the sham diet^*^
	Sham diet CD, *n* = 12 UC, *n* = 13	Select fruits and grains with no impact on intakes of nutrients, fibre and FODMAPS [suitable carbohydrates include fruits [eg, apples, bananas, and pears] and grains [eg, wheat]	Restriction: similar number of staple and non-staple foods to low FODMAP diet [eg, oranges, raspberries, strawberries, and grains such as rice]	Placebo sham diet with an exclusion of similar intensity and burden to low FODMAP diet	As above	As above	As above	Compliance**:** 100%	As above
Fritsch 2021^40^	Low fat diet UC, *n* = 18	High consumption: fibre	Dietary plan: 10% kcal fat, 1– 5% kcal saturated fat, 5–9% kcal unsaturated fat with omega 6:3 ratio ~3:1 or lower	Fat-reduced diet combined with increased fibre intake may reduce colonic inflammation	1 ASA-24 2 Nutrihand [web-based food diary platform]	Baseline and end of wash-out period	1 ASA-24 [https://asa24ncinihgov/] 2 Nutrihand [http://wwwnutrihandcom]	↓ total fat [saturated and unsaturated fat]^*^ ↓ omega 6:3 ratio^*^ ↑ fibre intake^*^ Adherence: 87%	↓ total fat [saturated and unsaturated fat]^*^ ↓ omega 6:3 ratio ^*^ ↑ fibre^*^ ↑ sugar^*^ compared with iSAD
	Improved standard American diet UC, *n* = 18	Dietary plan: 35–40% kcal fat, 11–10% kcal saturated fat, 25–29% unsaturated fat with omega 6:3 ratio ~20–30:1	Not applicable	High fat content consistent with a Western dietary pattern as a comparator	As above	As above	As above	↓ refined sugar^*^ ↑ arachidonic acid^*^ ↑ fibre^*^ Adherence: 85%	As above
Zhang 2020^44^	Mediterranean diet CD, *n* = 26	High consumption: plant-based food and fibre (eg, green leafy vegetables, whole fruits, and legumes [≥6 servings/day]); fatty fish [100–200 g servings/week] and extra-virgin olive oil	Restriction: 10% kcal fat; added sugar and alcohol; red meat [1 serving/week], processed meat and food additives [maltodextrins and emulsifiers]	Diet based on finding from epidemiological studies that show association between disease incidence and dietary components	1 3-day weighed food records	Baseline and then monthly for 12 weeks	1 ESHA Food Analysis Software Version 113 X 2 ESHA Food Processor Nutrition Analysis Software	↑ whole grains and vegetables intake^*^ ↓ red and processed meat	↓ red and processed meat intake compared with a diversified diet
	Diversified diet [Mediterranean habitual diet] CD, *n* = 32	High consumption: plant-based food	Restriction: red and processed meat	Not applicable	As above	As above	As above	Red and processed meat intake remained stable	As above
Lewis 2021^41^	Mediterranean diet CD, *n* = 92	High consumption: fruits, vegetables and legumes; fibre grains, fermented dairy, olive oil and fish	Restriction: red and processed meat; alcohol [5-15 g/week]	Anti-inflammatory effects	1 24-h dietary recall 2 AMeD 3 HEI-2015 [diet quality]	Baseline, Weeks 3, 6, 9, and 12 [end of trial]	1 AMeD score 2 HEI-2015 score	↑ AMeD score at Week 6^*^ ↑ fruits and vegetables consumption at Week 6^*^ Improved diet quality^*^ Adherence: 64% [Week 6] and 42% [Week 12]	AMeD score at baseline and Week 6 was comparable to SCD Diet quality improved from baseline to Week 6 to a similar extent
	Specific carbohydrate diet CD, *n* = 99	High consumption: select fruits and vegetables; fibre	Exclusion: complex carbohydrates including disaccharide and polysaccharides (eg, grains [wheat, rye, oats, rice, quinoa, barley, corn] and dairy [lactose]); certain fruits and vegetables [canned or starchy] Restriction: processed meat, alcohol, carbonated beverages, juices	Undigested carbohydrates may promote bacterial overgrowth and overproduction of mucus, leading to intestinal lesions	As above	As above	As above	↑ fruits and vegetables consumption at Week 6^*^ Improved diet quality^*^ Adherence: 68% [Week 6] and 40% [Week 12]	As above
Strauss 2023^50^	Mediterranean diet UC, *n* = 22	Not reported	Restriction: sulphur-rich foods, beverages, food additives [eg, carrageenan], beverages high in sulphate/sulphur and sulphur-containing supplements [eg, chondroitin sulphate]	Modulating dietary sulphur intake through this diet pattern may impact markers of inflammation and gut microbiome	1 ASA-24 Canada 2 MDS [diet quality]	Baseline and Week 8 [end of trial]	1 PREvencion con DIetaMEDiterranea 14-item Mediterranean Diet Adherence Screener [modified]	↓ sulphur intake^*^	No difference in sulphur intake or MDS score
	Habitual diet UC, *n* = 18	Not applicable		Not applicable	As above		As above	No difference	As above
Haskey 2023^49^	Mediterranean diet UC, *n* = 15	High consumption: fruits, vegetables, full-fat dairy products wholegrain cereals, legumes, fatty fish, MUFA-rich fat [olive oil, avocado, nuts]	Restriction: red and processed meat, refined grains, processed baked goods, sweets, soft drinks and fresh juices, fast foods, and pre-cooked meals, omega-6 PUFAs [corn, safflower, sunflower oil, margarine, prepared salad dressing and highly processed foods]	Anti-inflammatory effects and evidence of increased abundance of favourable bacteria	1 ASA-24 Canada-2016 2 MDSS 3 HEI-2015	Baseline, Weeks 3, 6, 9, and 12 [end of trial]	1 Dietary Reference Intakes 2 MD diet pyramid	↑ MDSS score at Week 12 [increased adherence; ↑ fruits, vegetables, dairy products, legumes, olive oil and fish [MUFA], and ↓ sweets]^*^ ↑ HEI-2015 score [increased diet quality from ↑ fruit, vegetable and ↓ added sugar intake]^*^	↑ MDSS score at Week 12 [better adherence as evidenced by ↑ fruits and olive oil and ↓ sweets]^*^ ↑ HEI-2015 score^*^
	Canadian Habitual Diet UC, *n* = 13	Not applicable	Not applicable	Not applicable	As above	As above	As above	Not assessed	As above
Shabat 2021^43^	Ulcerative Colitis Exclusion Diet UC, *n* = 15	Mandatory consumption: certain fruits and vegetables [tryptophan, natural sources of pectin and resistant starch] Prescribed amount: chicken, eggs, and yoghurt Increased exposure to tryptophan and natural sources of pectin and resistant starch	Restriction: sulphated amino acids, total protein, haeme, beef, animal fat, saturated fat, dairy fat, and food additives	Altered dietary components that may adversely affect goblet cells, mucus permeability, and microbiome composition in UC	1 Modified MARS diet adherence questionnaire 2 Dietitian assessment by direct questioning	Weeks 2 and 8	Not applicable	Adherence: 100% [Week 2] and 100% [Week 8]	Not assessed
Olendzki 2022^51^	IBD-AID CD, *n* = 13 UC, *n* = 7	High consumption: prebiotics: fruits, vegetables, oats, and honey; probiotics: fermented foods [no dairy], fermented dairy foods; beneficial foods: fatty acids [MUFAs and omega- 3, PUFAs] vegetable protein and lean animal protein	Exclusion: adverse foods: trans-fatty acids, processed and ultra processed foods [eg, processed fried animal protein, fried vegetable protein, artificial sweeteners, and high sugar beverages] Restriction: adverse foods: saturated fats [eg, high fat animal protein], wheat, lactose, selected gluten-free grains, corn and starchy vegetables, and selected condiments [wheat-based soy sauce, high in fructose corn sugar, containing carrageenan, maltodextrin or/and emulsifiers]	Increased consumption of foods rich in prebiotics and probiotics while avoiding food that trigger symptoms may revert dysbiosis in IBD	1 24-h IBD-AID Food Queries	3 times per week for 8 weeks	Not applicable	Not applicable	↑ all prebiotics*, ↑ probiotics [fermented diary products*] ↑ beneficial food consumption [omega-3*] ↓ adverse foods consumption^*^ [except artificial sweeteners] ↑ oats and ↓ selected avoided condiments in UC^*^ ↑ vegetable protein and ↓ processed fried animal protein, corn, starchy vegetables in CD^*^
	Habitual diet CD, *n* = 13 UC, *n* = 7	Not applicable	Not applicable	Not applicable	As above	3 times per week for 6 weeks [baseline]	Not applicable	↑ lean animal protein in CD^*^ Similar diets; ↓ intake of fruits and vegetables [<2 servings; comparable to average American diet]	As above
Sahu 2021^42^	Standard-of-care diet UC, *n* = 30	Dietary plan: Normal diet with dietary counselling about calorie and protein requirement	Not applicable	Not applicable	Charted by dietitian	7 days	1 Average daily calorie and protein intake was calculated by dividing the total calorie and protein consumption by 7	Not assessed	Average daily calorie [1264 kcal] and protein [42 g/d] intakes were comparable to EEN [1421 kcal and 50 g/d, respectively]

FODMAP, fermentable oligosaccharides, disaccharides, monosaccharides, and polyols; GOS, galacto-oligosaccharide; kcal, kilocalorie; g, grams; d, day; wk, week; ASA-24, Automated Self-Administered 24-h dietary recall; WD, Western diet; AMeD, Alternate Mediterranean Diet Score; MUFA, monounsaturated fatty acid; PUFA, polyunsaturated fatty acid; HEI-2015, Healthy Eating Index 2015; MDS, Mediterranean Diet Score; MDSS, Mediterranean Diet Serving Score; MARS, Medication Adherence Rating Scale; IBD-AID, Inflammatory Bowel Disease-Anti-Inflammatory Diet; EEN, exclusive enteral nutrition.

^*^Indicates statistically significant p values [*p* <005].

The diet protocol inclusion and restrictions highlight that some dietary modifications centred around the restriction of specific dietary components, and others were focused on the complete exclusion of single or multiple food groups. Most studies [*n* = 7 of 9] implemented increased intakes of fruits, vegetables, and fibre and restricted consumption of certain meats such as red and processed meats. Five studies reduced the intake of specific dietary fats^40,43,44,49,51^ [eg, animal fat, saturated and unsaturated fat, and omega 6:3 ratio], and four of these studies restricted consumption of food additives such as emulsifiers.^43,44,49,51^ The impact of a 12-week Mediterranean-inspired diet, which is chiefly characterised by increased intake of plant-based foods and limited consumption of red and processed meat, was investigated by Zhang *et al*.,^44^ Lewis *et al*.,^41^ and Haskey *et al*.^49^ All three studies reported significant increase in the consumption of fruits and vegetables from baseline to end of trial in IBD participants, with Lewis *et al*. and Haskey *et al.* also noting significant improvement in diet quality and adherence in the MD groups compared with the habitual diet groups. Similar results were observed in the SCD arm as the MD arm by Lewis *et al*.;, however, the adherence rates for both diets were insufficient [64% and 68%, respectively].

By contrast, Strauss *et al*.^50^ trialled an 8-week MD diet with a special focus on reducing sulphur intake in UC patients, but found no significant differences in sulphur consumption and diet quality between the MD group and those consuming a habitual diet. A 4-week low-fat diet by Fritsch *et al.*^40^ led to a significant reduction in total fat [saturated and unsaturated] and omega 6:3 ratio in UC patients, and had a similar high adherence rate [87%] as the comparator iSAD group [85%]. Similarly, Cox *et al*.^39^ reported high compliance with a 4-week low FODMAP diet [88%] in UC patients, as well as significant reductions in FODMAP, protein, fat, and sugar intake compared with a sham diet. The UCED diet used by Shabat *et al*.,^43^ which restricted total protein and fat [especially saturated fats] from animal sources as well as food additives, demonstrated the highest adherence rate [100%] after 8 weeks. Olendzki *et al*.^51^ trialled an 8-week IBD-AID diet in UC patients which also involved limiting consumption of ‘adverse foods’ such as animal protein and fat, and food additives from processed foods, while increasing intake of ‘beneficial foods’ eg, fruits, vegetables, and omega-3. This study did not measure diet adherence or quality, but a significant increase in consumption of prebiotics, probiotics, and ‘beneficial foods’, and decrease in consumption of ‘adverse foods’ according to the diet protocol [Table 3], was reported at the end of the trial compared with baseline [habitual diet].

Although studies investigating enteral nutrition was an exclusion criterion in the literature search process, Sahu *et al*.^42^ was included in this review due to the comparator group receiving standard-of-care IBD diet. The SOC diet involved a normal whole-food diet coupled with dietary counselling about calorie and protein intake, which was found to be comparable to exclusive enteral nutrition [EEN] in UC patients; however, corticosteroid failure occurred in more patients in the EEN group compared with SOC.

### 3.5. Changes in GI symptoms and quality of life

Only two of the nine intervention studies evaluated GI symptoms using a range of validated tools [Table 4]. Lewis *et al.*^41^ reported significant symptom improvement in CD patients on both the MD and SCD diets from screening to Week 6, but this change did not significantly differ between the treatment arms. In contrast, Cox *et al*.^39^ showed that a low FODMAP diet was significantly more effective in facilitating symptom relief, with reductions in bloating and flatulence severity and daily stool frequency, compared with the sham [low FODMAP] diet. This improvement in symptoms was defined by the reduction in IBS severity scoring system, and when patients were stratified by IBD type, this was more prominent in UC .

**Table 4.** Summary of findings: changes in GI symptoms and quality of life following dietary intervention

First author, year	Diet type	GI symptom assessment method	Quality of life assessment	Data collection time points	Changes in GI symptoms and quality of life			
					Baseline versus end oftrial	Intervention group versus comparator group		
					Within group	CD/UC	CD	UC
Cox 2020^39^	Low FODMAP diet	1. IBS-SSS 2. Global Symptom Question 3. GSRS	1. UK-specific IBD questionnaire	End of trial [Week 4]	↓ score for bloating severity ^*^ ↓ total IBS-SSS score compared with sham	↓ bloating severity^*^ ↑ achieved 50% IBS-SSS reduction^*^ ↑ adequate relief^*^ ↓ flatulence severity^*^ ↓ daily stool frequency^*^ ↑ total IBD questionnaire [better HR QOL]^*^	No difference	↑ reduction in IBS-SSS score compared with sham and end-of-trial^*^
	Sham diet	As above	As above	As above	No difference	No difference	No difference	No difference
Fritsch 2021^40^	Low fat diet	Not assessed	1. SIBDQ 2. SF-36	Baseline and end of was- out period	Improved QOL, role limitations owing to physical activity, emotional health, social functioning, bodily pain, and general health^*^	↑ QoL than iSAD^*^	Not assessed	Improved QoL^*^
	Improved standard American diet	Not assessed	As above	As above	As above	Improved QoL^*^	Not assessed	Improved QoL^*^
Lewis 2021^41^	Specific carbohydrate diet	1. sCDAI 2. CDAI 3. RAPID-3 4. BASFI	1. SIBDQ 2. PROMIS	Weeks 6 and 12 [end of trial]	Improvement in sCDAI, CDAI, sIBDQ, fatigue, sleep interference, pain, and social isolation^*^	Not assessed	No difference	Not assessed
	Mediterranean diet	As above	As above	As above	As above	Not assessed	No difference	Not assessed
Haskey 2023^49^^a^	Mediterranean diet	Not assessed	SIBDQ	Baseline, Weeks 3, 6, 9, and 12	↓ bowel symptoms [reduction in passing of gas and improvement in tenesmus*]	Not applicable	Not applicable	No difference
	Canadian Habitual Diet	Not assessed	As above	As above	No difference	Not applicable	Not applicable	As above
Zhang 2020^44^	Mediterranean diet	Not assessed	Not assessed	Not applicable	Not assessed	Not assessed	Not assessed	Not assessed
	Diversified diet [Mediterranean]	Not assessed	Not assessed	Not applicable	Not assessed	Not assessed	Not assessed	Not assessed
Shabat, 2021^43^	Ulcerative Colitis Exclusion Diet	Not assessed	Not assessed	Not applicable	Not assessed	Not assessed	Not assessed	Not assessed

IBS-SSS, Irritable Bowel Syndrome Severity System Scores; GSRS, GI Symptom Rating scale; N/A, not applicable; SIBDQ, Short Inflammatory Bowel Disease Questionnaire; SF-36, Short Form-36 Health Survey; QoL, quality of life; sCDAI, short Crohn’s Disease Activity Index; CDAI, Crohn’s Disease Activity Index; PROMIS, Patient-Reported Outcomes Management Information System; RAPID-3, Routine Assessment of Patient Index Data 3; BASFI, Bath Ankylosing Spondylitis Functional Index.

^a^Results taken from previously published work using the same cohort of participants.

^*^Indicates statistically significant *p*-values [*p* <0.05].

These same two studies^39,41^ and Fritsch *et al*.^40^ also reported on the impact of diet on the quality of life [QoL] of patients using a variety of validated methods [Table 4]. Lewis *et al*.^41^ showed that both the MD and SCD diets resulted in a significant QoL improvement in a cohort of CD patients at the end of the trial compared with baseline. However, no difference was seen when the two groups were compared. The iSAD diet investigated by Fritsch *et al.* also improved the overall QoL in IBD patients, but to a lesser extent than its low-fat diet comparator. When stratified by IBD subtypes, this effect was observed in UC but not in the CD group. The MD diet^49^ also led to an improvement in QoL, represented by a significant relief in some bowel symptoms of UC patients when compared with the habitual diet group. The low FODMAP diet used by Cox *et al*.,^39^ but not the sham diet, also led to better health-related quality of life [HR-QoL] when CD and UC were analysed together, but not separately.

### 3.6. Changes in clinical activity and inflammatory markers

To measure the effect of diet on disease activity, researchers mainly used scores such as the Harvey-Bradshaw [HBI], Partial Mayo Score [PMS], IBD Control Questionnaire, and Simple Clinical Colitis Activity Index [SCCAI], and objective markers such as C-reactive protein [CRP] and faecal calprotectin [FC] [Table 5]. Notably, eight out of nine studies used FC testing with a significant reduction in FC concentration only being observed in UC patients who were on EEN^42^ and in CD patients who were on an SCD diet.^41^ Similarly, most studies also measured CRP and found no difference in levels following the diets except for the UC patients in the EEN group, where a reduction in CRP concentration was observed after just 5 days of the 7-day trial.

**Table 5:** Summary of findings: changes in clinical disease activity and inflammatory markers following dietary intervention.

First author, year	Diet type	Disease activity assessment method	Inflammatory marker assessment method	Data collection time points	Changes in clinical disease activity and inflammatory markers			
					Baseline versus end of trial	Intervention versus comparator [between groups]		
					Within groups	CD/UC	CD	UC
Cox 2020^39^	Low FODMAP diet	1. HBI 2. PMS 3. IBD Control Questionnaire	1. FC 2. CRP	Baseline and Week 4 [end of trial]	No difference	No difference	No difference	↑ patient-perceived control of IBD
	Sham diet	As above	As above	As above	No difference	No difference	No difference	No difference
Fritsch 2021^40^	Low fat diet	PMS	1. FC 2. Serum CRP 3. Cytokines [IL1β, TNF-α, SAA, INF-γ]	Baseline and end of wash-out period	↓ SAA^*^	Not applicable	Not applicable	↓ SAA^*^
	Improved standard American diet	As above	As above	As above	No difference	Not applicable	Not applicable	No difference
Lewis 2021^41^	Specific Carbohydrate diet	sCDAI	1. FC 2. hsCRP	Weeks 6 and 12 [end of trial]	No difference	Not applicable	↓ FC concentration^*^	Not applicable
	Mediterranean diet	As above	As above	As above	No difference	Not applicable	No difference	Not applicable
Zhang 2020^44^	Mediterranean diet	Not assessed	FC	Baseline, Weeks 4, 8, and 12	No difference	Not applicable	No difference	Not applicable
	Diversified diet [Mediterranean]	As above	As above	As above	No difference	Not applicable	No difference	Not applicable
Haskey 2023^49^	Mediterranean diet	1. SCCAI 2. PMS	1. FC 2. Serum CRP 3. Serum albumin 4. Faecal sIgA	Baseline and Week 12	↑ faecal sIgA concentration^*^	Not applicable	Not applicable	No difference
	Canadian habitual diet	As above	As above	As above	↑ Partial Mayo Score^*^ ↑ SCCAI score^*^ [loss of clinical response] ↑ FC concentration^*^	Not applicable	Not applicable	As above
Strauss 2023^50^	Mediterranean diet	PMS	FC	Baseline and Week 8	Improved PMS score	Not applicable	Not applicable	No difference
	Habitual diet	As above	As above	As above	Improved PMS score	Not applicable	Not applicable	As above
Olendzki 2022^51^	IBD-AID	Not assessed	1. Cytokines [GM-CSF, IL-1I β, IL-2, IL-4, IL-5, IL-6, IL-8, IL-10, IL-12p70, 1L-13, IL-17A, IL-23, IFN- γ, TNF- α]	Week 14 [end of trial]	No difference	No difference	Not applicable	Not applicable
	Habitual diet	As above	As above	Week 1 [baseline]	No difference	No difference	Not applicable	Not applicable
Shabat 2021^43^	Ulcerative Colitis Exclusion diet	1. SCCAI 2. Mayo score	1. FC 2. CRP	Baseline, Weeks 8 and 12	↓ SCCAI^*^ ↓ Mayo score^*^	Not applicable	Not applicable	↑ endoscopic remission [Week 8] ↑ mucosal healing [Mayo 0] ↓ worsening of disease Compared with UCED plus FT and free diet plus FT groups
Sahu 2021^42^	Standard of care [normal diet]	Not assessed	1. FC 2. CRP 3. Serum albumin	Baseline to Day 7	↓ serum albumin [Day 7]^*^	Not applicable	Not applicable	↑ median CRP [Day 5]^*^ ↓ CRP reduction [Day 5]^*^ ↓ fall in FC concentration^*^ Compared with EEN group

HBI, Harvey-Bradshaw Index; PMS, Partial Mayo Score; FC, faecal calprotectin; CRP, C-reactive protein; IL1β, interleukin 1 beta; TNF-α, tumour necrosis factor alpha; SAA, serum amyloid A; INF-γ, interferon gamma; sIgA, secretory immunoglobulin A; GM-CSF, granulocyte-macrophage colony-stimulating factor; sCDAI, short Crohn’s Disease Activity Index; hsCRP, high sensitivity C-reactive protein; SCCAI, Simple Clinical Colitis Activity Index.

^*^Indicates statistically significant *p-*values [*p *<0.05].

Cox *et al*.^39^ did not display any changes in disease activity in any of the parameters measured other than the UC subgroup demonstrating a greater patient-perceived control of IBD following a low FODMAP diet. Levels of pro-inflammatory cytokines were quantified by Fritsch *et al*.^40^ and Olendzki *et al*.^51^; however, neither study detected significant differences in the concentrations of any of the cytokines measured following the LFD and the IBD-AID, respectively, in UC patients. Fritsch *et al*. only reported a significant reduction of serum amyloid-A [SAA] concentration in UC patients on an LFD at baseline versus. end of trial, as well as compared with iSAD.

### 3.7. Microbiota analysis methods

Overall, 12 out of 13 studies evaluated the intestinal microbiota from stool, two studies examined the intestinal tissue [inflamed and non-inflamed from the ileo-rectal region], and one study^48^ investigated both specimen types [Table 6]. Most of the studies implemented commercial extraction kits according to the manufacturer’s instructions for DNA extraction, but two studies^39,42^ followed protocols established elsewhere. The sample collection kits used varied between studies, and samples were stored mostly at -80°C or at -20°C. Eight studies^40,42,44,45,47–50^ assessed the intestinal microbiota by 16S rRNA amplicon gene sequencing which targeted the V3-V4 variable regions, except for one study^46^ that used the V5-V6 regions. The remaining studies^39,41,43,51^ employed metagenomic shotgun sequencing [MGS]. Analysis pipelines and reference databases used to assess taxonomy were also highly variable between studies.

**Table 6:** Characteristics of included studies investigating intestinal microbiota profiles of IBD patients and healthy controls.

First author, year	Specimen type	Tissue site	Collection time points	Sample collection and storage	Extraction method	Profiling approach	Platform	Variable regions amplified	Analysis pipeline and taxonomic database
Cox 2020^39^	Stool	Not applicable	Baseline and end of trial	Not reported; ice then, -80°C	IHMS SOP 07 V2 protocol	Shotgun	Ion Proton Sequencer	Not applicable	Quantitative metagenomic pipeline NCBI database [V Nov 2016]
Lewis 2021^41^	Stool	Not applicable	Baseline, Weeks 6 and 12	Not reported	DNeasy PowerSoil kit [Qiagen]	Shotgun	Illumina HiSeq	Not applicable	Sunbeam pipeline^52^ Kraken
Shabat 2021^43^	Stool	Not applicable	Pre-baseline	Sterile container with an anaerobic generator bag [Anaerogen]; -80°C	DNeasy PowerMag Soil kit [Qiagen]	Shotgun	Illumina NovaSeq	Not applicable	Bowtie^53^ Segata Lab SGB^11^
Olendzki 2022^51^	Stool	Not applicable	Baseline and end of trial	OMNIgene Gut collection kit [Genotek]; - 80°C	MagAttract PowerSoil DNA Kit [Qiagen]	Shotgun	NextSeq 500	Not applicable	MetaPhlan2 V2.9.14 ChocoPhlAn
Weng 2020^48^	Stool and biopsy	Colorectum; inflamed and non-inflamed	Single time point	Stool: sterile 1.5-mL tube; not reported Biopsy: sterile cryovials containing RNAlater solution [Qiagen]; -80°C	DNA Extraction Kit [Qiagen]	16S	Illumina MiSeq	V4	QIIME 1.9.1 pipeline NCBI databanks^54^
Fritsch 2021^40^	Stool	Not applicable	Baseline and end of trial	EasySampler stool collection kit; -4°C, then -80°C	PowerSoil/faecal DNA Isolation Kit [Qiagen]	16S	Illumina MiSeq	V4	CosmosID pipeline CosmosID curated database
Strauss 2023^50^	Stool	Not applicable	Baseline and week 8	Not reported; -20°C, then -80 °C	PowerSoil Pro DNA kit [Qiagen]	16S	Illumina MiSeq	V4	UPARSE pipeline The Ribosomal Database Project
Haskey 2023^49^	Stool	Not applicable	Baseline and Week 12	Sterile 50-mL polypropylene conical tubes; -80°C	QIAamp Powerfaecal Pro DNA kit [Qiagen]	16S	Illumina MiSeq	V4	QIIME 2 Greengenes V 13.8
Sahu 2021^42^	Stool	Not applicable	Baseline and end of trial	Not reported; -80°C	THSTI protocol	16S	Illumina MiSeq	V3-V4	QIIME 2 V 2020.2 pipeline Greengenes 13.8 database
Zhang 2020^44^	Stool	Not applicable	Baseline, Weeks 4, 8, and 12	Stored in home freezer; -80°C	AquaStool preservative reagent [MultiTarget Pharmaceuticals]	16S	Illumina Miseq	V3-V4	QIIME 2 pipeline Silva database
Teofani 2022^47^	Stool	Not applicable	Single time point	PSP stool collection tubes [Invitek Molecular]; not reported	PSP Spin Stool DNA Kit Plus [Invitek Molecular]	16S	Illumina MiSeq	V3-V4	QIIME 2 Silva database [V 138]
Berbisá 2022^45^	Stool	Not applicable	Single time point	OMNIgene Gut Stool Microbiome Kit [Genotek]; - 20 °C	DNeasy PowerSoil Pro kit [Qiagen]	16S	Illumina MiSeq	V3-V4	Not reported
Schreiner 2019^46^	Biopsy	Ileum, rectum; inflamed and non-inflamed	Single time point	2-mL microfuge tubes containing RNAlater [Sigma-Aldrich]; -20°C	Prep DNA/RNA Mini Kit [Qiagen]	16S	IonTorrent Personal Genome Machine	V5-V6	QIIME 1.9.1 pipeline Greengenes database

IHMS SOP, International Human Microbiome Standards Standard Operating Procedure; NCBI, National Centre for Biotechnology Information; THSTI, Translational Health Sciences and Technology Institute; QIIME, Quantitative Insights Into Microbial Ecology; SGB, Species-level Genome Bins; PSP, Pre-analytical Sample Processing.

### 3.8. Changes in diversity indices and relative abundances

All nine trials characterised the intestinal microbiota using stool samples and reported on alpha diversity [within sample variation] either by richness, observed species, Shannon, Simpson, Faith’s phylogenetic diversity, or Pielou’s evenness [Supplementary Table S7]. However, no significant shifts were detected within or between any of the groups following diet intervention. Of note, Shabat *et al.*^43^ did not perform microbiota sequencing of stool from UC patients who received either the UCED diet alone or the UCED or free diet in combination with FT. Instead, stool from healthy donors was analysed before and after conditioning with a specific diet for 2 weeks, but no changes in alpha diversity were observed. All four cross-sectional studies measured alpha diversity either by richness, Shannon, Simpson, Chao1, or observed operational taxonomic units [OTUs], and only two studies yielded significant differences. Weng *et al.*^48^ demonstrated a significantly lower Shannon index score in CD patients consuming their habitual diet compared with the HC group. Teofani *et al*.^47^ also found significantly reduced alpha diversity, as measured by Shannon, Simpson, and Chao1, in IBD patients compared with HC consuming a habitual diet.

Beta diversity [across sample variation] was evaluated in all studies either by Bray-Curtis, Jaccard, Aitchison, Manhattan and Gower, or weighted or unweighted Unifrac. Statistical differences in beta diversity matrices were evaluated using either PERMANOVA or Mann-Whitney testing. Zhang *et al*.^44^ reported a significant difference in beta diversity between a habitual diversified diet [similar to an MD] and a non-diversified diet at baseline. However, after CD patients with a non-diversified diet had followed a 12-week MD diet, this difference was no longer evident. Haskey *et al.*^49^ also reported a significant shift in microbial composition between MD and CHD over time. Schreiner *et al*.^46^ noted significant differences in beta diversity between IBD patients who ate a vegetarian, gluten-free, or regular diet. Finally, Teofani *et al.*^47^ demonstrated reduced beta diversity in the IBD population consuming a normal diet compared with HC.

Changes in relative microbial abundance were measured in 11 of the 13 included studies [Supplementary Tables S5 and S6]. Cox *et al*.^39^ analysed stool samples at the end of a low FODMAP diet and only recorded a significant decrease in *Faecalibacterium prausnitzii* and changes in three *Bifidobacterium* species, namely a decrease in *Bifidobacterium adolescentis* and *dentium* and an increase in *Bifidobacterium longum;* however, these significant differences were not evident at the genus level. Similarly, Lewis *et al*.^41^ also observed a reduction in *Faecalibacterium prausnitzii* following an SCD diet intervention compared with an MD diet, and noted an increase in the family Enterobacteriaceae and lower *Eubacterium eligens* and *rectale.* Haskey *et al*.^49^ found that species primarily belonging to the phylum Firmicutes, including *Ruminococcus*, *Flavonifractor*, *Clostridium*, *Lactococcus,* and *Blautia* A spp., were most positively associated with an MD diet. The most negatively associated species belonged to a mixture of phyla including Firmicutes, Actinobacteria, and Bacteriodota, with *Bifidobacterium*, *Blautia*, *Veillonella*, *Streptococcus,* and *Massilioclostridium* spp. being the most abundant. It is worth noting that Strauss *et al*.^50^ also examined changes in microbial taxa; however, the profiles were associated with changes in FC levels rather than relating to the MD diet.

The low-fat diet by Fritsch *et al*.^40^ identified significant alterations in the faecal microbiota of UC participants at the phylum level, with an enrichment of Bacteroidetes and depletion of Actinobacteria reported after a LFD, whereas no changes in these phyla were observed with iSAD. Higher *Prevotella* and *Faecalibacterium prausnitzii* were also reported after an LFD compared with iSAD. The anti-inflammatory diet by Olendzki *et al*.^51^ led to increased abundance of SCFA-producing bacteria belonging to Clostridia class at the species level, with *Roseburia hominis* being the most dominant in the stool samples of all IBD subjects, as well as in CD participants only. UC patients exhibited high abundances of *Faecalibacterium prausnitzii*, *Eubacterium eligens*, *Coprococcus come,* and multiple *Bacteroides spp*., whereas overall, *Parabacteroides distasonis* was consistently depleted in all IBD participants during the IBD-AID intervention. Conversely, Sahu *et al*.^42^ only yielded significant increases in the genera *Bifidobacterium* and *Anaerosinus*, and a decline in *Catenibacterium* in the faecal samples of UC patients who received SOC relative to those who were on EEN. In the study by Zhang *et al*.,^44^ no significant microbial differences were found in the stool of CD patients eating a habitual diversified diet or a non-diversified diet when compared with their respective baseline samples. At baseline, significantly higher Proteobacteria [*Escherichia Shigella*] and *Paraprevotella*, and lower *Faecalibacterium* were reported in the diversified diet group compared with its counterpart. However, the opposite trend was observed in the non-diversified diet groups after being prescribed a Mediterranean-inspired diet at Weeks 4 and 12.

Of the cross-sectional studies, Schreiner *et al.*^46^ identified significantly lower mucosal abundance of Barnesiellaceae family, Clostridiales order, and genera within the phylum Firmicutes such as *Ruminococcus* and *Faecalibacterium prausnitzii*, in IBD participants who were gluten-free compared with those consuming a regular diet. Representative genera from Bacteroidetes were also reduced in the gluten-free CD participants. By contrast, vegetarian UC patients had a higher abundance of Firmicutes including *Blautia*, *Coprococcus*, *Dorea,* and *Ruminococcus*, whereas *Faecalibacterium prausnitzii* was found to be lower when compared with meat-eating [regular diet] participants. When comparing CD and UC patients on a normal diet with HC, Teofani *et al*.^47^ only reported significantly higher levels of faecal Atopobiaceae and Enterobacteriaceae in IBD, whereas Berbisá *et al*.^45^ found significantly increased abundance of families and genera belonging to the phylum Verrucomicrobia, such as Akkermansiaceae and Veillonellaceae, in the stool of UC participants. Of the included studies, Weng *et al*.^48^ identified the highest number of differentially abundant taxa [primarily at the class, order, and family levels] in the microbiota of IBD patients and HC, using a combination of mucosal and faecal samples. This study found significant enrichment of numerous bacteria belonging mainly to the phyla Proteobacteria, Actinobacteria, Acidobacteria, Deinococcota, Deferribacterota, and Fusobacteria, in both the CD and the UC patient samples, whereas HC only displayed a high abundance of Firmicutes. When sample source was considered, Weng *et al.*^48^ found that there was a significantly more abundant mucosa-associated microbiota compared with the faecal communities in both UC and CD patient samples.

## 4. Discussion

The current systematic review of 13 studies indicated inconsistent associations between diet and microbiota in IBD. Overall, this study collated information from a total of 1775 IBD participants who were on a habitual diet and 469 IBD participants who received a predefined dietary intervention. The primary feature of the types of diets trialled focused on the reduction of foods suspected of triggering inflammation, such as animal fat, red and processed meat, alcohol, and food additives [eg, emulsifiers and artificial sweeteners], with an emphasis on increased intake of fresh fruits and vegetables, and fibre.^40,41,43,44^ Others excluded carbohydrates responsible for fuelling microbial overgrowth and inducing functional symptoms.^39,41^ Of the nine intervention studies, only four studies demonstrated positive changes in either GI symptoms or QoL. Cox *et al*.^39^ assessed the impact of a low FODMAP diet on IBD patients with persistent GI symptoms, which met the Rome III criteria for IBS and found improvement in symptom scores and QoL, which is consistent with previous studies.^55,56^ However, a potential limitation of this study is the inability to differentiate between IBD and IBS-associated mediators of non-specific symptoms. As there is a high co-prevalence of IBD and IBS,^57^ dietary changes may be alleviating IBS rather than IBD symptoms; therefore measures of disease severity are needed. Lewis *et al*.^41^ demonstrated improvement in symptoms with both the SCD and MD diets, but neither diet was superior over the other in inducing symptomatic remission in CD patients. This may be due to similarities between the interventions, namely the emphasis on fresh fruits and vegetables, and the ratio of monounsaturated to polyunsaturated fatty acid consumption. The beneficial effect of an MD diet is supported by Haskey *et al*.^49^ through the achievement of reduced bowel symptoms in UC patients following the intervention. Fritsch *et al*.^40^ also reported improvement in clinical symptoms and QoL with a low-fat diet, and to a lesser extent awith n improved Western diet [iSAD diet], in UC patients. However, a higher fibre intake was also reinforced in both diets, and therefore, it is unclear whether these responses were a product of a low-fat or a high-fibre content. Dietary components that have been reported to prevent or induce IBD get confused with factors that may assist in management. Findings from this review indicate the potential for diet modulation to substantially improve symptoms and QoL.

Remarkably, a UC-specific exclusion diet by Shabat *et al*.^43^ induced endoscopic remission and mucosal healing [regarded as the optimal outcome in IBD research]^58^ in patients, which was highest among participants who were on the diet alone compared with those who either followed an exclusion or habitual diet but also underwent FT. The impact of this diet on the microbiota composition of these patients would have been enlightening, but this study only characterised the microbiota profile of the healthy faecal donors at pre-baseline and not the UC patients, which is a major limitation of this work. Moreover, the pre-conditioning diet provided to the donors was different from the exclusion diet prescribed to the UC patients, so it is difficult to draw conclusions regarding its efficacy. In concert, findings by Cox *et al*.^39^ and Shabat *et al*.^43^ suggest that complete dietary exclusion of specific food groups may have a more potent effect than a mere reduction of intake. However, improvements in clinical symptoms and QoL following a low-fat diet by Fritsch *et al*.^40^ indicate that this may be dependent on the type of dietary component restricted.

With regards to the intestinal microbiota, none of the intervention or observational studies exhibited changes in alpha diversity metrics post-diet, and a significantly lower beta diversity was only observed in two of the four cross-sectional studies investigating habitual diets in IBD patients compared with non-IBD HCs. In agreement with findings from another trial in IBS patients,^59^ Cox *et al*.^39^ also reported increased abundance of faecal *Bifidobacteria longum* and *adolescentis* and lower total *Faecalibacterium prausnitzii* following a low FODMAP diet compared with sham diet. Lewis *et al*.^41^ also reported decreased *Faecalibacterium prausnitzii* abundance in response to SCD.

Although SCD and low FODMAP dietary approaches have distinct principles, both diets target dietary carbohydrates. A low FODMAP diet excludes specific fermentable carbohydrates, whereas the more restrictive SCD prohibits consumption of disaccharides and complex carbohydrates known to support the growth of beneficial bacteria like *Faecalibacterium prausnitzii*.^60^ The reduction in dietary substrate with either low FODMAP or SCD may account for the decrease in their population compared with the other diets that showed an increase in *Faecalibacterium prausnitzii* abundance. Halmos *et al*.^61^ have shown a dysbiotic pattern in quiescent CD patients, whereby the relative abundances of butyrate-producing *Clostridium* cluster XIVa [phylum Firmicutes] and mucus-associated *Akkermansia muciniphila* [phylum Verrucomicrobia] were lower and the mucus-degrading *Ruminococcus torques* higher, in the low FODMAP diet compared with a typical Australian diet.^61,62^

This is further corroborated by Png *et al*.,^63^ and together suggests that a restricted FODMAP intake may promote an unfavourable GI tract environment and predispose the intestinal mucosa to inflammation. Whereas a low FODMAP diet is recommended for managing individuals with IBS, the efficacy of this treatment in patients with quiescent IBD with concurrent IBS-like symptoms is controversial and is yet to established. Overall, four studies^41,44,49,50^ featured the MD, which broadly appears to have positive associations with bacteria within the dominant Firmicutes phylum, such as *Faecalibacterium*, *Clostridium,* and *Blautia*, consistently found to be significantly depleted in the gut of IBD patients,^8,64^ indicating that diet is imperative in shifting the intestinal microbial dysbiosis towards a balanced community.

**Figure 2:**
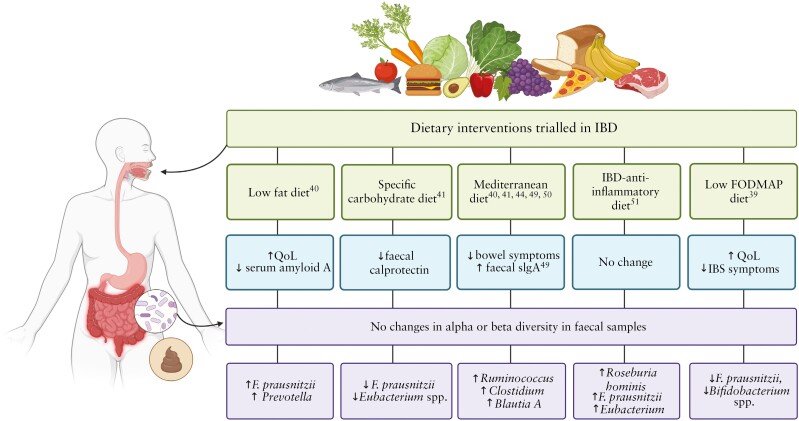
Schematic representation, summarising significant changes in symptoms or quality of life [blue] and microbiota diversity and relative abundances [purple] found in IBD patients post-dietary intervention [green] trial. IBD, inflammatory bowel disease; FODMAP, fermentable oligosaccharides, disaccharides, monosaccharides, and polyols; QoL, quality of life; sIgA, serum immunoglobulin A; IBS, irritable bowel syndrome. Created with BioRender.com.

In a similar vein, Olendzki *et al*.^51^ also showed that the IBD-AID can also favour these commonly diminished Firmicutes, including potent butyrate-producing species *Roseburia hominis*, *Faecalibacterium prausnitzii,* and *Eubacterium eligens*, which have been reported to have an anti-inflammatory capacity.^65,66^ In line with previous studies, Berbisá *et al*.^45^ reported reduced abundance of mucus-fortifying bacteria *Akkermansia* in UC patients compared with healthy subjects; however, it is uncertain whether this is a cause or consequence of disease.^67,68^ The absence of *Akkermansia* may be due to decreased mucins in UC patients.^69^ Recent research demonstrated that oral supplementation of *Akkermansia muciniphila* in mice counteracted the low-grade intestinal inflammation and alterations in species composition that are otherwise induced by consumption of the emulsifiers carboxylmethylcellulose [CMC] and polysorbate 80 [P80].^70^ Degradation of colonic mucin as a primary energy source by this bacterium results in the synthesis of SCFAs such as propionate and acetate,^67^ which have anti-inflammatory effects and are vital substrates for maintaining intestinal barrier integrity.^71^ Propionate and acetate, along with butyrate, are also produced during anaerobic bacterial fermentation of non-digestible carbohydrates such as dietary fibre and resistant starch.^31^ In IBD patients, a significant depletion in dominant butyrate-producing bacteria such as *Faecalibacterium prausnitzii* and *Roseburia intestinalis* has been noted.^64,72^ Furthermore, SCFA concentrations may be associated with disease activity, as adult patients with active IBD have been found to have significantly reduced levels of butyric, acetic, and propionic acids in the faeces compared with healthy subjects.^65,73^ Given the ability of these SCFAs to influence the intestinal microbiota composition and function, investigation into metabolomics in future studies is needed. A schematic representation of significant changes relating to symptoms or QoL, and microbiota diversity and relative abundance found in IBD participants, based on dietary interventions is provided [Figure 2].

This systematic review has notable limitations that warrant discussion. First, the retrieved studies were substantially heterogeneous in terms of design, participants, dietary intervention type, duration, and outcomes, which prohibited a meta-analysis. The relatively small sample size of the included studies makes it challenging to draw generalisable conclusions from results. Poor dietary data reporting was highly prevalent, culminating from the lack of unified standards to assess dietary intake. Inadequate recording of background and diet information throughout the study periods further affected our ability to describe changes attributed to diet. Given that the study location of the included studies was divided between North America, Northern Europe, and Asia, regional diet variations are another potential source of heterogeneity in findings, adding to the layers of complexity and challenge in IBD research. Variations in culture, dietary beliefs and practices, culinary preferences, and food availability and accessibility, have an impact on dietary patterns based on geographical location. Although elements of the Western diet are most prevalent in North America [USA and Canada] and Northern Europe [UK and Denmark], there can be significant variations within countries. For example, an American diet consists of larger portions and excess sugar compared with the UK,^74^ and other parts of Northern Europe, such as the Faroe Islands [Denmark], include a mix of traditional foods [eg, dried fish, whale, and blubber] within their diet.^45^ In contrast to Western diets, which often emphasise wheat-based products [eg, bread and pasta], red meat, and dairy, Asian diets typically incorporate rice as dietary staple as well as a greater range of plant-based foods [eg, vegetables, tofu, soy products, and spices].^75,76^ Food preparation techniques also differ between regions, wherein roasting, frying, and baking are more prevalent in Western countries whereas Asian populations tend to prefer cooking methods like steaming and stir-frying, which can greatly influence food composition and content.^76,77^ Regional diet variations are therefore important to consider in IBD research, as they not only impede the identification of consistent dietary triggers, but can also influence the degree to which individuals adhere to a specific dietary pattern or intervention, and lead to variabilities in baseline composition of the gut microbiota.^78^

The area of microbiome research is rapidly evolving. As a result, there are limitations to the methodological approaches undertaken in this field. To date, 16S rRNA-based methods have been more commonly employed to analyse the microbiota, and this was used in eight of the 13 studies included in this review. However, due to the lack of species-level sensitivity of 16S amplicon sequencing, future studies should focus on using whole metagenome sequencing techniques to evaluate the effect of diet on individual species. The origin of microbial sampling is also important to consider for intestinal microbiota profiling, as faecal samples cannot accurately reveal the alterations of intestinal microbiota, whereas biopsy samples provide a more precise reflection of mucosa-associated microbiota.^79^ As such, this review is limited by the fact that only two of the 13 studies examined the microbiota using tissue samples. Seven^40,44,45,47–49,51^ of the 12 studies that examined faecal microbiota used samples obtained using at-home self-collection kits provided to participants, and four studies^39,41–43^ did not specify how stool samples were collected. One study^50^ required participants to undergo baseline and end-of-trial endoscopic assessment, and although stool specimens were collected at these time points, it was unclear whether these were spontaneously passed or stool aspirate samples. Microbial profiles from aspirate samples can vary significantly from faecal profiles from the sample individual,^79,80^ and therefore is important to consider the type of stool specimen analysed when interpreting and comparing study results. Furthermore, the great variability in the sample collection and extraction methods used, as well as the analysis pipelines and taxonomic databases referred to between the studies, may also impact on the outcomes. The duration of a dietary intervention is another limiting factor that can possibly explain some of the difficulty in detecting compositional changes in the microbiota, with the longest interval being described as 12 weeks,^41,43,44,49^ and as short as 1 week in one study.^42^

Whereas direct effects of diet on the microbiota are likely observable within days,^4^ short-term dietary changes lead to immediate but transient shifts which may not necessarily reflect sustained improvements in IBD symptoms. The type and intensity of a dietary intervention can also determine the extent to which the microbiota is altered, as studies have demonstrated that some diets, such as those with substantial modification in fibre content, macronutrient ratio, or ‘beneficial foods’ [eg, prebiotics, probiotics, or fermented foods], may have more profound effects on certain microbial populations.^4,81^ Longer interventions, spanning weeks to months, are likely required for observing significant and stable responses of the microbiota to a dietary intervention, and in turn, microbiome-mediated effects on host disease, phenotype, or biomarkers.^82^ Consequently, this study highlights the considerable potential for future research in diet-microbiota interactions in IBD.

## 4.1. Conclusion

This systematic review suggests that current evidence regarding the modulatory effect of a specific dietary pattern, intervention, or constituents, on the intestinal microbiota and disease course of IBD patients is rather limited. The included studies were highly variable and changes in the diversity and abundance of intestinal microbial communities in IBD patients post-diet intervention, were minimal. Dietary patterns including the low FODMAP and low fat diets were associated with improvements in disease severity and QoL, but these were not necessarily associated with microbiota profiles, and therefore conclusions regarding diet-microbiota interactions in IBD are limited. Greater consistency in dietary and microbiota assessment methods and future studies that focus on similar interventions are necessary to identify signatures and predict diet-induced microbiota responses. Understanding precisely how to modulate the microbiota composition, metabolism, and function through a dietary approach could allow the development of personalised IBD dietary recommendations.

## Supplementary Data

Supplementary data are available at *ECCO-JCC* online.

jjad204_suppl_Supplementary_Tables_S1-S6

jjad204_suppl_Supplementary_Table_S7
