# Supplementary material for: The Impact of Dietary Interventions on the Microbiota in Inflammatory Bowel Disease: A Systematic Review
Source: J Crohns Colitis. 2023 Dec 15;18(6):920–42. doi: 10.1093/ecco-jcc/jjad204 (PMC11147801; doi:10.1093/ecco-jcc/jjad204)
Supplement: jjad204_suppl_Supplementary_Tables_S1-S6 [file jjad204_suppl_supplementary_tables_s1-s6.docx]

**Supplementary Materials**

**Supplementary Table S1.** The Preferred Reporting Items for Systematic Reviews and Meta-Analyses (PRISMA) Checklist

| **Section and Topic** | **Item #** | **Checklist item** | **Location where item is reported** |
| --- | --- | --- | --- |
| **TITLE** | | |  |
| Title | 1 | Identify the report as a systematic review. | 1 |
| **ABSTRACT** | | |  |
| Abstract | 2 | See the PRISMA 2020 for Abstracts checklist. | 2-3 |
| **INTRODUCTION** | | |  |
| Rationale | 3 | Describe the rationale for the review in the context of existing knowledge. | 4-5 |
| Objectives | 4 | Provide an explicit statement of the objective(s) or question(s) the review addresses. | 6 |
| **METHODS** | | |  |
| Eligibility criteria | 5 | Specify the inclusion and exclusion criteria for the review and how studies were grouped for the syntheses. | 7 & Supplementary Table S2 |
| Information sources | 6 | Specify all databases, registers, websites, organisations, reference lists and other sources searched or consulted to identify studies. Specify the date when each source was last searched or consulted. | 7 & Supplementary Table S3 |
| Search strategy | 7 | Present the full search strategies for all databases, registers and websites, including any filters and limits used. | 7 & Supplementary Table S3 |
| Selection process | 8 | Specify the methods used to decide whether a study met the inclusion criteria of the review, including how many reviewers screened each record and each report retrieved, whether they worked independently, and if applicable, details of automation tools used in the process. | 8 |
| Data collection process | 9 | Specify the methods used to collect data from reports, including how many reviewers collected data from each report, whether they worked independently, any processes for obtaining or confirming data from study investigators, and if applicable, details of automation tools used in the process. | 8 |
| Data items | 10a | List and define all outcomes for which data were sought. Specify whether all results that were compatible with each outcome domain in each study were sought (e.g. for all measures, time points, analyses), and if not, the methods used to decide which results to collect. | N/A |
|  | 10b | List and define all other variables for which data were sought (e.g. participant and intervention characteristics, funding sources). Describe any assumptions made about any missing or unclear information. | N/A |
| Study risk of bias assessment | 11 | Specify the methods used to assess risk of bias in the included studies, including details of the tool(s) used, how many reviewers assessed each study and whether they worked independently, and if applicable, details of automation tools used in the process. | 9 & Supplementary Table S4 |
| Effect measures | 12 | Specify for each outcome the effect measure(s) (e.g. risk ratio, mean difference) used in the synthesis or presentation of results. | N/A |
| Synthesis methods | 13a | Describe the processes used to decide which studies were eligible for each synthesis (e.g. tabulating the study intervention characteristics and comparing against the planned groups for each synthesis (item #5)). | N/A |
|  | 13b | Describe any methods required to prepare the data for presentation or synthesis, such as handling of missing summary statistics, or data conversions. | N/A |
|  | 13c | Describe any methods used to tabulate or visually display results of individual studies and syntheses. | N/A |
|  | 13d | Describe any methods used to synthesize results and provide a rationale for the choice(s). If meta-analysis was performed, describe the model(s), method(s) to identify the presence and extent of statistical heterogeneity, and software package(s) used. | N/A |
|  | 13e | Describe any methods used to explore possible causes of heterogeneity among study results (e.g. subgroup analysis, meta-regression). | N/A |
|  | 13f | Describe any sensitivity analyses conducted to assess robustness of the synthesized results. | N/A |
| Reporting bias assessment | 14 | Describe any methods used to assess risk of bias due to missing results in a synthesis (arising from reporting biases). | N/A |
| Certainty assessment | 15 | Describe any methods used to assess certainty (or confidence) in the body of evidence for an outcome. | N/A |
| **RESULTS** | | |  |
| Study selection | 16a | Describe the results of the search and selection process, from the number of records identified in the search to the number of studies included in the review, ideally using a flow diagram. | Figure 1 |
|  | 16b | Cite studies that might appear to meet the inclusion criteria, but which were excluded, and explain why they were excluded. | Figure 1 |
| Study characteristics | 17 | Cite each included study and present its characteristics. | Table 1 |
| Risk of bias in studies | 18 | Present assessments of risk of bias for each included study. | 10 & Supplementary Table S4 |
| Results of individual studies | 19 | For all outcomes, present, for each study: (a) summary statistics for each group (where appropriate) and (b) an effect estimate and its precision (e.g. confidence/credible interval), ideally using structured tables or plots. | N/A |
| Results of syntheses | 20a | For each synthesis, briefly summarise the characteristics and risk of bias among contributing studies. | 10 |
|  | 20b | Present results of all statistical syntheses conducted. If meta-analysis was done, present for each the summary estimate and its precision (e.g. confidence/credible interval) and measures of statistical heterogeneity. If comparing groups, describe the direction of the effect. | N/A |
|  | 20c | Present results of all investigations of possible causes of heterogeneity among study results. | N/A |
|  | 20d | Present results of all sensitivity analyses conducted to assess the robustness of the synthesized results. | N/A |
| Reporting biases | 21 | Present assessments of risk of bias due to missing results (arising from reporting biases) for each synthesis assessed. | N/A |
| Certainty of evidence | 22 | Present assessments of certainty (or confidence) in the body of evidence for each outcome assessed. | N/A |
| **DISCUSSION** | | |  |
| Discussion | 23a | Provide a general interpretation of the results in the context of other evidence. | 20 - 24 |
|  | 23b | Discuss any limitations of the evidence included in the review. | 23 - 25 |
|  | 23c | Discuss any limitations of the review processes used. | 23 |
|  | 23d | Discuss implications of the results for practice, policy, and future research. | 24 - 26 |
| **OTHER INFORMATION** | | |  |
| Registration and protocol | 24a | Provide registration information for the review, including register name and registration number, or state that the review was not registered. | 7 |
|  | 24b | Indicate where the review protocol can be accessed, or state that a protocol was not prepared. | N/A |
|  | 24c | Describe and explain any amendments to information provided at registration or in the protocol. | N/A |
| Support | 25 | Describe sources of financial or non-financial support for the review, and the role of the funders or sponsors in the review. | N/A |
| Competing interests | 26 | Declare any competing interests of review authors. | 28-29 |
| Availability of data, code and other materials | 27 | Report which of the following are publicly available and where they can be found: template data collection forms; data extracted from included studies; data used for all analyses; analytic code; any other materials used in the review. | N/A |

*From:*  Page MJ, McKenzie JE, Bossuyt PM, Boutron I, Hoffmann TC, Mulrow CD, et al. The PRISMA 2020 statement: an updated guideline for reporting systematic reviews. BMJ 2021;372:n71. doi: 10.1136/bmj.n71

For more information, visit: <http://www.prisma-statement.org/>

**Supplementary Table S2.** Eligibility Criteria Following the PICOS Approach for Inclusion of Studies

| **PICOS** | **Inclusion and exclusion criteria** | **Data extracted** |
| --- | --- | --- |
| Participants or population | **Inclusion:** Adult human populations ≥  18 years with IBD (Crohn’s disease  or ulcerative colitis)  **Exclusion:** Children ≤ 18 years, animal  studies | Age, gender, IBD subtype, disease  activity, disease location,  gastrointestinal symptoms |
| Intervention or exposure | **Inclusion:** Studies that reported on the  participants’ diet (using a dietary  intervention or habitual diet) and  examined microbiota composition  (16S or MGS) were included.  **Exclusion:** Studies with no dietary  data and employed other  microbiota profiling methods | Type of diet (intervention or habitual  diet), dietary assessment tools,  duration of diet, number of  participants, mean or median intake  and range  Specimen type, collection timepoints,  DNA extraction method, profiling  approach, variable regions  amplified, taxonomic database and  taxonomic target |
| Comparison or intervention | **Inclusion:** Comparator was a standard  or sham diet, habitual diet, or  different diet either in IBD or  healthy cohorts. There were no  limits on the number of comparison  groups.  **Exclusion:** Studies solely investigating  EEN | Number and description of control  group such as age and gender  matching |
| Outcomes | One or more dietary outcomes  including energy, macronutrients,  fibre, diet quality or dietary patterns.  Changes in the GI microbiota  composition and symptoms/disease  outcomes with diet. | Dietary intake, intake compared to  Controls or recommended  requirements  Changes in the GI microbiome  composition (alpha diversity, beta  diversity and relative abundances)  Comparison of end point data within or  between groups |
| Study design | **Inclusion:** Cross-sectional, cohort and  longitudinal and interventional  studies (i.e., RCT).  **Exclusion:** Case-controls and other  study designs | Type of study design, location of study,  study duration |

IBD, inflammatory bowel disease; MGS, metagenomic shotgun sequencing; EEN, exclusive enteral nutrition; GI, gastrointestinal; RCT, randomised controlled trial.

**Supplementary Table S3**. Search Strategy

| **Search #** | **MEDLINE Search** |
| --- | --- |
| 1 | exp Microbiota/ |
| 2 | microbiota.ti,ab,kw,kf. |
| 3 | microbiome.ti,ab,kw,kf. |
| 4 | mycobiome.ti,ab,kw,kf |
| 5 | periphyton.ti,ab,kw,kf. |
| 6 | virome.ti,ab,kw,kf. |
| 7 | microbial consorti*.ti,ab,kw,kf. |
| 8 | 16S.ti,ab,kw,kf. |
| 9 | shotgun.ti,ab,kw,kf. |
| 10 | metagenomic*.ti,ab,kw,kf. |
| 11 | 1 or 2 or 3 or 4 or 5 or 6 or 7 or 8 or 9 or 10 |
| 12 | exp Inflammatory Bowel Diseases/ |
| 13 | inflammatory bowel disease*.ti,ab,kw,kf. |
| 14 | crohn*.ti,ab,kw,kf. |
| 15 | ulcerative colitis.ti,ab,kw,kf. |
| 16 | (colitis or enteritis or ileitis or enterocolitis).ti,ab,kw,kf. |
| 17 | IBD.ti,ab,kw,kf. |
| 18 | 11 or 12 or 13 or 14 or 15 |
| 19 | exp Diet/ |
| 20 | nutrition*.ti,ab,kw,kf. |
| 21 | food*.ti,ab,kw,kf. |
| 22 | (diet* adj3 (therap* or regimen* or eliminat* or exclu* or intervention*)).ti,ab,kw,kf. |
| 23 | (diet* adj1 (fodmap* or anti-inflammatory or carbohydrate or vegetarian or semi-vegetarian or gluten-free or paleo* or Mediterranean or fibre or fiber)).ti,ab,kw, kf. |
| 24 | 17 or 18 or 19 or 20 or 21 |
| 25 | 10 and 16 and 22 |
| 26 | limit 23 to yr="2013 -Current" |
| 27 | limit 26 to english language |
| **Search #** | **EMBASE Search** |
| 1 | exp microflora/ |
| 2 | microbiota.ti,ab,kw. |
| 3 | microbiome.ti,ab,kw. |
| 4 | mycobiome.ti,ab,kw. |
| 5 | periphyton.ti,ab,kw. |
| 6 | virome.ti,ab,kw. |
| 7 | microbial consorti*.ti,ab,kw. |
| 8 | 16S.ti,ab,kw. |
| 9 | shotgun.ti,ab,kw. |
| 10 | metagenomic*.ti,ab,kw. |
| 11 | 1 or 2 or 3 or 4 or 5 or 6 or 7 or 8 or 9 or 10 |
| 12 | exp inflammatory bowel disease/ |
| 13 | inflammatory bowel disease*.ti,ab,kw. |
| 14 | crohn*.ti,ab,kw. |
| 15 | ulcerative colitis.ti,ab,kw. |
| 16 | (colitis or enteritis or ileitis or enterocolitis).ti,ab,kw. |
| 17 | IBD.ti,ab,kw. |
| 18 | 12 or 13 or 14 or 15 or 16 or 17 |
| 19 | exp Diet/ |
| 20 | nutrition*.ti,ab,kw. |
| 21 | food*.ti,ab,kw. |
| 22 | (diet* adj3 (therap* or regimen* or eliminat* or exclu* or intervention*)).ti,ab,kw. |
| 23 | (diet* adj1 (fodmap* or anti-inflammatory or carbohydrate or vegetarian or semi-vegetarian or gluten-free or paleo* or Mediterranean or fibre or fiber)).ti,ab,kw. |
| 24 | 19 or 20 or 21 or 22 or 23 |
| 25 | 11 or 18 or 24 |
| 26 | limit 23 to yr="2013 -Current" |
| 27 | limit 26 to english language |
| 28 | limit 27 to conference abstract |
| 29 | 27 not 28 |
| **Search #** | **Web of Science Search** |
| 1 | microbiota OR microbiome OR mycobiome OR periphyton OR virome OR "microbial consort*" OR 16S OR shotgun OR metagenomic* (Topic) |
| 2 | inflammatory bowel disease* OR crohn* OR "ulcerative colitis" OR IBD OR colitis OR enteritis OR ileitis OR enterocolitis (Topic) |
| 3 | diet OR nutrition* OR food* OR (diet* NEAR/3 (therap* OR regimen* OR eliminat* OR exclu* OR intervention*)) OR (diet* NEAR/1 (fodmap* OR "anti-inflammatory" OR carbohydrate OR vegetarian OR "semi-vegetarian" OR "gluten-free" OR paleo* OR Mediterranean OR fibre OR fiber)) (Topic) |
| 4 | Review Articles or Articles (Document Types) and English (Languages) |
| 5 | Timespan: 2013-01-01 to 2023-06-07 (Publication date) |
| 6 | #1 AND #2 AND #3 AND # AND #5 |
| **Search #** | **Scopus Search** |
| 1 | (TITLE-ABS-KEY) (microbiota OR microbiome OR mycobiome OR periphyton OR virome OR "microbial consort*" OR 16S OR shotgun OR metagenomic*) |
| 2 | AND (TITLE-AS-KEY ("inflammatory bowel disease*" OR crohn* OR "ulcerative colitis" OR IBD OR colitis OR enteritis OR ileitis OR enterocolitis) |
| 3 | AND (TITLE-ABS-KEY (diet OR nutrition* OR food* OR (diet* W/3 (therap* OR regimen* OR eliminat* OR exclu* OR intervention*)) OR (diet* W/1 (fodmap* OR "anti-inflammatory" OR carbohydrate OR vegetarian OR "semi-vegetarian" OR "gluten-free" OR paleo* OR Mediterranean OR fibre OR fiber))) |
| 4 | AND PUBYEAR > 2013 LANGUAGE (english) AND (LIMIT-TO (DOCTYPE, "ar") OR (LIMIT-TO (DOCTYPE, "re")) |
| **Search #** | **Cochrane Library Search** |
| 1 | MeSH descriptor: [Microbiota] explode all trees |
| 2 | MeSH descriptor: [RNA, Ribosomal, 16S] |
| 3 | (microbiome OR mycobiome OR periphyton OR virome OR "microbial consort*" OR shotgun OR metagenomic*):ti,ab,kw (Word variations have been searched) |
| 4 | #1 OR #2 OR #3 |
| 5 | MeSH descriptor: [Inflammatory Bowel Diseases] explode all trees |
| 6 | ("inflammatory bowel disease*" OR crohn* OR "ulcerative colitis" OR IBD OR colitis OR enteritis OR ileitis OR enterocolitis):ti,ab,kw (Word variations have been searched) |
| 7 | #5 OR #6 |
| 8 | MeSH descriptor: [Diet] explode all trees |
| 9 | diet OR nutrition* OR food* OR (diet* NEAR/3 (therap* OR regimen* OR eliminat* OR exclu* OR intervention*)) OR (diet* NEAR/1 (fodmap* OR "anti-inflammatory" OR carbohydrate OR vegetarian OR "semi-vegetarian" OR "gluten-free" OR paleo* OR Mediterranean OR fibre OR fiber)) |
| 10 | #8 OR #9 (Word variations have been searched) |
| 11 | #4 AND #7 AND #10 with Cochrane Library publication date Between Jan 2013 and June 2022 |

**Supplementary Table S4.** Risk of Bias Assessment According to the JBI Critical Appraisal Tools for RTCs, Quasi-experimental and Cross-sectional Studies

| **Randomised controlled trials** | | | | | | | | |  | | | | | | |
| --- | --- | --- | --- | --- | --- | --- | --- | --- | --- | --- | --- | --- | --- | --- | --- |
| **Reference** | **Q1** | **Q2** | **Q3** | **Q4** | **Q5** | **Q6** | **Q7** | **Q8** | | **Q9** | **Q10** | **Q11** | **Q12** | **Q13** | **Score** |
| Cox *et al*., 2020^39^ | Y | Y | Y | Y | N | U | Y | Y | | Y | Y | Y | Y | Y | 11/13 |
| Fritsch *et al*., 2021^40^ | Y | N | Y | Y | U | U | Y | Y | | N | Y | Y | Y | Y | 9/13 |
| Lewis *et al*., 2021^41^ | Y | Y | Y | N | N | N | Y | Y | | Y | Y | Y | Y | Y | 10/13 |
| Sahu *et al*., 2021^42^ | Y | Y | Y | N | N | N | Y | Y | | Y | Y | Y | Y | Y | 10/13 |
| Shabat *et al*., 2021^43^ | Y | Y | Y | N | Y | U | N | Y | | Y | Y | U | Y | Y | 9/13 |
| Haskey *et al*., 2023^49^ | Y | U | Y | Y | N | U | Y | Y | | Y | Y | Y | Y | Y | 10/13 |
| Strauss *et al*., 2023^50^ | Y | Y | Y | U | N | U | Y | Y | | N | Y | U | Y | Y | 8/13 |

Y = Yes, N = No, U = Unclear

**Key: Q1:** Was true randomisation used for assignment of participants to treat groups? Q2: Was allocation to treatment groups concealed? Q3: Were treatment groups similar at baseline? Q4: Were participants blind to the treatment assignment? Q5: Were those delivering treatment blind to treatment assignment? Q6: Were outcome assessors blind to treatment assignment? Q7: Were treatment groups treated identically other than the intervention of interest? Q8: Was follow up complete and if not, were differences between groups in terms of their follow up adequately described and analysed? Q9: Were participants analysed in the groups to which they were randomised? Q10: Were outcomes measured in the same way for treatment groups? Q11:Were outcomes measured in a reliable way? Q12: Was appropriate statistical analysis used? Q13: Was the trial design appropriate, and any deviations from the standard RCT design accounted for in the conduct and analysis of the trial?

| **Quasi-experimental study** | | | | | | | | | | | | | |
| --- | --- | --- | --- | --- | --- | --- | --- | --- | --- | --- | --- | --- | --- |
| **Reference** | **Q1** | **Q2** | **Q3** | **Q4** | **Q5** | **Q6** | **Q7** | **Q8** | **Q9** | **Score** |  |  |  |
| Zhang *et al*., 2020^44^ | Y | Y | N | Y | Y | Y | Y | U | Y | 7/9 |  |  |  |
| Olendzki *et al*., 2022^51^ | Y | Y | Y | N | Y | Y | Y | U | Y | 7/9 |  |  |  |

Y = Yes, N = No, U = Unclear

**Key:** Q1: Is it clear in the study what is the ‘cause’ and what is the ‘effect’? Q2: Were the participants included in any comparisons similar? Q3: Were the participants included in any comparisons receiving similar treatment/care, other than the exposure or intervention of interest? Q4: Was there a control group? Q5: Were there multiple measurements of the outcome both pre and post the intervention/exposure? Q6: Was follow up complete and if not, were differences between groups in terms of their follow up adequately described and analysed? Q7: Were the outcomes of participants included in any comparisons measured in the same way? Q8: Were the outcomes measured in a reliable way? Q9: Was appropriate statistical analysis used?

| **Cross-sectional studies** | | | | | | | | | | | | | |
| --- | --- | --- | --- | --- | --- | --- | --- | --- | --- | --- | --- | --- | --- |
| **Reference** | **Q1** | **Q2** | **Q3** | **Q4** | **Q5** | **Q6** | **Q7** | **Q8** | **Score** |  |  |  |  |
| Schreiner *et al*., 2019^46^ | N | Y | U | U | N | N | U | Y | 2/8 |  |  |  |  |
| Weng *et al*., 2020^48^ | Y | Y | U | Y | N | N | Y | Y | 5/8 |  |  |  |  |
| Teofani *et al*., 2022^47^ | Y | Y | U | U | Y | Y | U | Y | 5/8 |  |  |  |  |
| Berbisá *et al*., 2022^45^ | Y | Y | Y | Y | Y | Y | U | Y | 7/8 |  |  |  |  |

Y = Yes, N = No, U = Unclear

**Key:** Q1: Were the criteria for inclusion in the sample clearly defined? Q2: Were the subjects and the setting described in detail? Q3: Was the exposure measured in a valid and reliable way? Q4: Were objective, standard criteria used for measurement of the condition? Q5: Were confounding factors identified? Q6: Were strategies to deal with confounding factors stated? Q7: Were the outcomes measured in a valid and reliable way? Q8: Was appropriate statistical analysis used?

**Supplementary Table S5.** Summary of Findings: Relative Abundance of the GIT Microbiota in IBD Patients and Healthy Controls

| **Study** | Cox *et al*., 2020^39^**^Ⴕ^** | Fritsch *et al*., 2021^40^**^Ⴕ^** | | | Lewis *et al*., 2021^41^ | Haskey *et al.* 2023^49^**^Ⴕ^** | Olendzki *et al*. 2022 ^51^**^Ⴕ^** | | | Sahu *et al*., 2021^42^**^Ⴕ^** | Zhang *et al*., 2020^44^ | | | | | Schreiner *et al*., 2019^46^ | | | Weng *et al*., 2020^48^**^Ⴕ^** | | | | | | | Teofani *et al*., 2022^47^ | Berbisá *et al*., 2022^45^**^Ⴕ^** |
| --- | --- | --- | --- | --- | --- | --- | --- | --- | --- | --- | --- | --- | --- | --- | --- | --- | --- | --- | --- | --- | --- | --- | --- | --- | --- | --- | --- |
| **Study details** | Low FODMAP diet  (CD/UC, *n* = 21)  vs.  Sham diet  (CD/UC, *n* = 22) | LFD  (UC, *n* = 17) [Baseline] | iSAD  (UC, *n* = 17)  [Baseline] | LFD  (UC *n* = 17)  vs.  iSAD  (UC, *n* = 17) | SDC (CD, *n* = 99) vs. Mediterranean diet (CD, *n* = 92) [End-of-trial] | MD (UC, n = 15) vs. CHD (UC, n = 13) [End-of-trial, wk 12] | IBD-AID (CD/UC, *n* = 19) vs. Habitual (baseline) (CD/UC, *n* = 22) | IBD-AID (CD, *n* = 12) vs. Habitual (baseline) (CD, *n* 12= 22) | IBD-AID (UC, *n* = 7) vs. Habitual (baseline) (UC, *n* = 7) | SOC (UC, *n* = 13) vs. EEN (UC, *n* = 14) [End-of-trial] | DD  (CD, *n* = 25)  [Baseline vs. end of trial, wk 12] | NDD  (CD, *n* = 15)  [Baseline vs. end of trial, wk 12] | DD  (CD, *n* = 25)  vs.  NDD  (CD, *n* = 15)  [Baseline] | DD  (CD, *n* = 25)  vs.  NDD  (CD, *n* = 15)  [wk 4] | DD  (CD, *n* = 25)  vs.  NDD  (CD, *n* = 15)  [End of trial, wk 12] | GFD (CD, *n* = 6) vs. regular diet (CD, *n* = 41) | GFD (UC, *n* = 6) vs. regular diet (UC, *n* = 25) | Vegetarian vs. (UC, *n* = 12) regular diet (UC, *n* = 25) | Habitual diet  (CD, *n* = 58) Combined biopsy and stool) | Habitual diet  (HC, *n* = 24)  Combined (biopsy and stool) | Habitual diet  (UC, *n* = 31)  Combined (biopsy and stool) | Habitual diet  (UC, *n* = 31)  Biopsy | Habitual diet  (UC, *n* = 31)  Stool | Habitual diet  (CD, *n* = 58)  Biopsy | Habitual diet  (CD, *n* = 58)  Stool | Habitual diet  (CD, *n* = 52; UC, *n* = 58) vs.  (HC, *n* = 42) | Habitual diet  (UC, *n* = 41)  vs.  (HC, *n* = 144) |
| **Phylum** | | | | | | | | | | | | | | | | | | | | | | | | | | | |
| Actinobacteria |  | **↓** | = | = |  |  |  |  |  |  | = | = |  |  |  |  |  |  |  |  |  |  |  |  |  |  |  |
| Bacteriodetes |  | **↑** | = | = |  |  |  |  |  |  | = | = |  |  |  | **↓** |  |  |  |  |  |  |  |  |  |  |  |
| Firmicutes |  |  |  |  |  |  |  |  |  |  |  |  |  |  |  | **↓** | **↓** | **↑** |  |  |  |  |  |  |  |  |  |
| Proteobacteria |  |  |  |  |  |  |  |  |  |  | = | = | **↑** | **↓** | **↓** |  |  |  |  |  |  |  |  |  |  |  |  |
| Verrucomicrobia |  |  |  |  |  |  |  |  |  |  |  |  |  |  |  |  |  |  |  |  |  |  |  |  |  |  | **↓** |
| **Class** | | | | | | | | | | | | | | | | | | | | | | | | | | | |
| Acidobacteria |  |  |  |  |  |  |  |  |  |  |  |  |  |  |  |  |  |  | **↑** |  |  | **↑** |  | **↑** |  |  |  |
| Acidobacteria_Gp3 |  |  |  |  |  |  |  |  |  |  |  |  |  |  |  |  |  |  |  |  |  |  |  | **↑** |  |  |  |
| Acidobacteria_Gp4 |  |  |  |  |  |  |  |  |  |  |  |  |  |  |  |  |  |  |  |  |  |  |  | **↑** |  |  |  |
| Acidobacteria_Gp6 |  |  |  |  |  |  |  |  |  |  |  |  |  |  |  |  |  |  |  |  |  | **↑** |  | **↑** |  |  |  |
| Bacilli |  |  |  |  |  |  |  |  |  |  |  |  |  |  |  |  |  |  | **↑** |  |  | **↑** |  |  |  |  |  |
| Bacteroidia |  | **↑** |  |  |  |  |  |  |  |  |  |  |  |  |  |  |  |  |  |  |  |  | **↑** |  | **↑** |  |  |
| Chlamydiales |  |  |  |  |  |  |  |  |  |  |  |  |  |  |  |  |  |  |  |  |  |  |  | **↑** |  |  |  |
| Chlamydiia |  |  |  |  |  |  |  |  |  |  |  |  |  |  |  |  |  |  |  |  |  | **↑** |  |  |  |  |  |
| Chloroplast |  |  |  |  |  |  |  |  |  |  |  |  |  |  |  |  |  |  | **↑** |  |  | **↑** |  |  |  |  |  |
| Clostridia |  |  |  |  |  |  |  |  |  |  |  |  |  |  |  |  |  |  |  | **↑** |  |  |  |  |  |  |  |
| Coriobacteriaceae |  |  |  |  |  |  |  |  |  |  |  |  |  |  |  |  |  |  |  |  |  |  |  | **↑** |  |  |  |
| Cyanobacteria |  |  |  |  |  |  |  |  |  |  |  |  |  |  |  |  |  |  |  |  |  |  |  | **↑** |  |  |  |
| Cytophagia |  |  |  |  |  |  |  |  |  |  |  |  |  |  |  |  |  |  |  |  |  | **↑** |  | **↑** |  |  |  |
| Deferribacteres |  |  |  |  |  |  |  |  |  |  |  |  |  |  |  |  |  |  |  |  |  |  |  | **↑** |  |  |  |
| Deinococci |  |  |  |  |  |  |  |  |  |  |  |  |  |  |  |  |  |  | **↑** |  |  | **↑** |  |  |  |  |  |
| Erysipelotrichia |  |  |  |  |  |  |  |  |  |  |  |  |  |  |  |  |  |  |  |  |  | **↑** |  |  |  |  |  |
| Fibrobacteria |  |  |  |  |  |  |  |  |  |  |  |  |  |  |  |  |  |  |  |  | **↑** |  |  |  |  |  |  |
| Flavobacteriia |  |  |  |  |  |  |  |  |  |  |  |  |  |  |  |  |  |  | **↑** |  |  | **↑** |  | **↑** |  |  |  |
| Fusobacteriia |  |  |  |  |  |  |  |  |  |  |  |  |  |  |  |  |  |  | **↑** |  |  |  |  |  |  |  |  |
| Lentisphaeria |  |  |  |  |  |  |  |  |  |  |  |  |  |  |  |  |  |  |  |  |  | **↑** |  |  |  |  |  |
| Methanobacteria |  |  |  |  |  |  |  |  |  |  |  |  |  |  |  |  |  |  |  |  |  |  |  | **↑** |  |  |  |
| Negativicutes |  |  |  |  |  |  |  |  |  |  |  |  |  |  |  |  |  |  |  |  |  |  | **↑** |  |  |  |  |
| Shingobacteriia |  |  |  |  |  |  |  |  |  |  |  |  |  |  |  |  |  |  |  |  | **↑** |  |  |  |  |  |  |
| Sphingobacteriia |  |  |  |  |  |  |  |  |  |  |  |  |  |  |  |  |  |  |  |  |  | **↑** |  |  |  |  |  |
| Sphinobacteriia |  |  |  |  |  |  |  |  |  |  |  |  |  |  |  |  |  |  |  |  |  |  |  | **↑** |  |  |  |
| Verrucomicrobiae |  |  |  |  |  |  |  |  |  |  |  |  |  |  |  |  |  |  |  |  |  |  |  |  |  |  | **↓** |
| **Order** | | | | | | | | | | | | | | | | | | | | | | | | | | | |
| Acidimicrobiales |  |  |  |  |  |  |  |  |  |  |  |  |  |  |  |  |  |  |  |  |  |  |  | **↑** |  |  |  |
| Actinomycetales |  | **↓** |  |  |  |  |  |  |  |  |  |  |  |  |  |  |  |  |  |  | **↑** | **↑** |  | **↑** |  |  |  |
| Bacillales |  |  |  |  |  |  |  |  |  |  |  |  |  |  |  |  |  |  | **↑** |  |  | **↑** |  |  |  |  |  |
| Bacteriodales |  | **↑** |  |  |  |  |  |  |  |  |  |  |  |  |  |  |  |  |  |  |  |  | **↑** |  | **↑** |  |  |
| Bifidobacteriales |  |  |  |  |  |  |  |  |  |  |  |  |  |  |  |  |  |  |  |  | **↑** |  |  | **↑** |  |  |  |
| Caulobacterales |  |  |  |  |  |  |  |  |  |  |  |  |  |  |  |  |  |  |  |  | **↑** | **↑** |  |  |  |  |  |
| Chlamydiales |  |  |  |  |  |  |  |  |  |  |  |  |  |  |  |  |  |  |  |  |  | **↑** |  |  | **↑** |  |  |
| Chloroplast |  |  |  |  |  |  |  |  |  |  |  |  |  |  |  |  |  |  |  |  |  |  |  |  | **↑** |  |  |
| Clostriadiales |  |  |  |  |  |  |  |  |  |  |  |  |  |  |  |  |  |  |  | **↑** |  |  |  |  |  |  |  |
| Clostridia_u_o |  |  | **↓** |  |  |  |  |  |  |  |  |  |  |  |  |  |  |  |  |  |  |  |  |  |  |  |  |
| Coriobacteriales |  |  |  |  |  |  |  |  |  |  |  |  |  |  |  |  |  |  |  |  |  | **↑** |  | **↑** |  |  |  |
| Cytophagales |  |  |  |  |  |  |  |  |  |  |  |  |  |  |  |  |  |  |  |  |  | **↑** |  | **↑** |  |  |  |
| Deferribacterales |  |  |  |  |  |  |  |  |  |  |  |  |  |  |  |  |  |  |  |  |  |  |  | **↑** |  |  |  |
| Deinococcales |  |  |  |  |  |  |  |  |  |  |  |  |  |  |  |  |  |  | **↑** |  |  | **↑** |  |  |  |  |  |
| Deinococci |  |  |  |  |  |  |  |  |  |  |  |  |  |  |  |  |  |  |  |  |  |  |  | **↑** |  |  |  |
| Erysipelotrichales |  |  |  |  |  |  |  |  |  |  |  |  |  |  |  |  |  |  |  |  |  | **↑** |  |  |  |  |  |
| Fibrobacterales |  |  |  |  |  |  |  |  |  |  |  |  |  |  |  |  |  |  |  |  | **↑** |  |  |  |  |  |  |
| Flavobacteriales |  |  |  |  |  |  |  |  |  |  |  |  |  |  |  |  |  |  | **↑** |  |  | **↑** |  | **↑** |  |  |  |
| Fusobacteriales |  |  |  |  |  |  |  |  |  |  |  |  |  |  |  |  |  |  | **↑** |  |  |  |  |  |  |  |  |
| Lactobacillales |  |  |  |  |  |  |  |  |  |  |  |  |  |  |  |  |  |  | **↑** |  |  | **↑** |  |  |  |  |  |
| Methanobacteriales |  |  |  |  |  |  |  |  |  |  |  |  |  |  |  |  |  |  |  |  |  |  |  | **↑** |  |  |  |
| Methylococcales |  | **↓** |  |  |  |  |  |  |  |  |  |  |  |  |  |  |  |  |  |  |  |  |  |  |  |  |  |
| Neisseriales |  | **↑** |  |  |  |  |  |  |  |  |  |  |  |  |  |  |  |  |  |  |  |  |  |  |  |  |  |
| Planctomycetia |  |  |  |  |  |  |  |  |  |  |  |  |  |  |  |  |  |  |  |  |  | **↑** |  |  |  |  |  |
| Pseudomonadales |  | **↑** |  |  |  |  |  |  |  |  |  |  |  |  |  |  |  |  |  |  |  |  |  |  |  |  |  |
| Rhizobiales |  |  |  |  |  |  |  |  |  |  |  |  |  |  |  |  |  |  | **↑** |  |  |  |  |  |  |  |  |
| Rhodobacterales |  |  |  |  |  |  |  |  |  |  |  |  |  |  |  |  |  |  |  |  | **↑** |  |  |  |  |  |  |
| Rhodospirillales |  |  |  |  |  |  |  |  |  |  |  |  |  |  |  |  |  |  |  |  | **↑** |  |  |  |  |  |  |
| Selenomonadales |  |  |  |  |  |  |  |  |  |  |  |  |  |  |  |  |  |  |  |  |  |  | **↑** |  |  |  |  |
| Sphingobacteriales |  |  |  |  |  |  |  |  |  |  |  |  |  |  |  |  |  |  |  |  | **↑** | **↑** |  | **↑** |  |  |  |
| Sphingomonadales |  |  |  |  |  |  |  |  |  |  |  |  |  |  |  |  |  |  |  |  | **↑** |  |  |  |  |  |  |
| Verrucomicrobiales |  |  |  |  |  |  |  |  |  |  |  |  |  |  |  |  |  |  | **↑** |  |  |  |  |  |  |  | **↓** |
| Victivallales |  |  |  |  |  |  |  |  |  |  |  |  |  |  |  |  |  |  |  |  |  | **↑** |  |  |  |  |  |
| **Family** | | | | | | | | | | | | | | | | | | | | | | | | | | | |
| Acidimicrobiaceae |  |  |  |  |  |  |  |  |  |  |  |  |  |  |  |  |  |  |  |  |  |  |  | **↑** |  |  |  |
| Actinomycetaceae |  |  |  |  |  |  |  |  |  |  |  |  |  |  |  |  |  |  |  |  |  | **↑** |  | **↑** |  |  |  |
| Aerococcaceae |  |  |  |  |  |  |  |  |  |  |  |  |  |  |  |  |  |  |  |  |  | **↑** |  |  |  |  |  |
| Akkermansiaceae |  |  |  |  |  |  |  |  |  |  |  |  |  |  |  |  |  |  |  |  |  |  |  |  |  |  | **↓** |
| Atopobiaceae |  |  |  |  |  |  |  |  |  |  |  |  |  |  |  |  |  |  |  |  |  |  |  |  |  | **↑** |  |
| Bacillaceae1 |  |  |  |  |  |  |  |  |  |  |  |  |  |  |  |  |  |  | **↑** |  |  | **↑** |  | **↑** |  |  |  |
| Bacillales_IncertaeSedisXI |  |  |  |  |  |  |  |  |  |  |  |  |  |  |  |  |  |  |  |  |  | **↑** |  | **↑** |  |  |  |
| Bacillales_IncertaeSedisXII |  |  |  |  |  |  |  |  |  |  |  |  |  |  |  |  |  |  | **↑** |  |  |  |  | **↑** |  |  |  |
| Bacteroidaceae |  |  |  |  |  |  |  |  |  |  |  |  |  |  |  |  |  |  |  |  |  |  | **↑** |  | **↑** |  |  |
| Bifidobacteriaceae |  |  |  |  |  |  |  |  |  |  |  |  |  |  |  |  |  |  |  |  | **↑** |  |  | **↑** |  |  |  |
| Bradyrhizobiaceae |  |  |  |  |  |  |  |  |  |  |  |  |  |  |  |  |  |  | **↑** |  |  | **↑** |  |  |  |  |  |
| Brevibacteriaceae |  |  |  |  |  |  |  |  |  |  |  |  |  |  |  |  |  |  | **↑** |  |  |  |  | **↑** |  |  |  |
| Brucellaceae |  |  |  |  |  |  |  |  |  |  |  |  |  |  |  |  |  |  | **↑** |  |  | **↑** |  |  |  |  |  |
| Carnobacteriaceae |  |  |  |  |  |  |  |  |  |  |  |  |  |  |  |  |  |  | **↑** |  |  | **↑** |  |  |  |  |  |
| Caulobacteraceae |  |  |  |  |  |  |  |  |  |  |  |  |  |  |  |  |  |  |  |  | **↑** | **↑** |  |  |  |  |  |
| Cellulomonadaceae |  |  |  |  |  |  |  |  |  |  |  |  |  |  |  |  |  |  |  |  |  |  |  | **↑** |  |  |  |
| Chitinophagaceae |  |  |  |  |  |  |  |  |  |  |  |  |  |  |  |  |  |  |  |  | **↑** | **↑** |  | **↑** |  |  |  |
| Chloroplast |  |  |  |  |  |  |  |  |  |  |  |  |  |  |  |  |  |  | **↑** |  |  | **↑** |  | **↑** |  |  |  |
| Clostridia_u_f |  |  | **↓** |  |  |  |  |  |  |  |  |  |  |  |  |  |  |  |  |  |  |  |  |  |  |  |  |
| Clostridiales_IncertaeSedisXI |  |  |  |  |  |  |  |  |  |  |  |  |  |  |  |  |  |  | **↑** |  |  | **↑** |  |  |  |  |  |
| Clostridiales_IncertaeSedisXIII |  |  |  |  |  |  |  |  |  |  |  |  |  |  |  |  |  |  |  | **↑** |  | **↑** |  |  |  |  |  |
| Clostridiaceae1 |  |  |  |  |  |  |  |  |  |  |  |  |  |  |  |  |  |  |  |  | **↑** |  |  |  |  |  |  |
| Coriobacteriaceae |  |  |  |  |  |  |  |  |  |  |  |  |  |  |  |  |  |  |  |  |  | **↑** |  | **↑** |  |  |  |
| Corynebacteriaceae |  |  |  |  |  |  |  |  |  |  |  |  |  |  |  |  |  |  |  |  | **↑** | **↑** |  | **↑** |  |  |  |
| Cryomorphaceae |  |  |  |  |  |  |  |  |  |  |  |  |  |  |  |  |  |  |  |  |  | **↑** |  | **↑** |  |  |  |
| Cytophagaceae |  |  |  |  |  |  |  |  |  |  |  |  |  |  |  |  |  |  |  |  |  | **↑** |  | **↑** |  |  |  |
| Deferribacteraceae |  |  |  |  |  |  |  |  |  |  |  |  |  |  |  |  |  |  |  |  |  |  |  | **↑** |  |  |  |
| Defluvitaleaceae |  |  |  |  |  |  |  |  |  |  |  |  |  |  |  |  |  |  | **↑** |  |  | **↑** |  |  |  | **↑** |  |
| Deinococcaceae |  |  |  |  |  |  |  |  |  |  |  |  |  |  |  |  |  |  | **↑** |  |  | **↑** |  | **↑** |  |  |  |
| Dermabacteraceae |  |  |  |  |  |  |  |  |  |  |  |  |  |  |  |  |  |  | **↑** |  |  | **↑** |  | **↑** |  |  |  |
| Dermacoccaceae |  |  |  |  |  |  |  |  |  |  |  |  |  |  |  |  |  |  |  |  |  |  |  | **↑** |  |  |  |
| Dietziaceae |  |  |  |  |  |  |  |  |  |  |  |  |  |  |  |  |  |  |  |  |  |  |  | **↑** |  |  |  |
| Enterobacteriaceae |  |  |  |  | **↑** |  |  |  |  |  |  |  |  |  |  |  |  |  |  |  |  |  |  |  |  |  |  |
| Enterococcaceae |  |  |  |  |  |  |  |  |  |  |  |  |  |  |  |  |  |  | **↑** |  |  | **↑** |  |  |  |  |  |
| Enterococcus |  |  |  |  |  |  |  |  |  |  |  |  |  |  |  |  |  |  | **↑** |  |  |  |  |  |  |  |  |
| Erysipelotrichaceae |  |  |  |  |  |  |  |  |  |  |  |  |  |  |  |  |  |  |  |  |  | **↑** |  |  |  |  |  |
| Eubacteriaceae |  |  |  |  |  |  |  |  |  |  |  |  |  |  |  |  |  |  |  |  |  | **↑** |  |  |  |  |  |
| Fibrobacteraceae |  |  |  |  |  |  |  |  |  |  |  |  |  |  |  |  |  |  |  |  | **↑** |  |  |  |  |  |  |
| Flavobacteriaceae |  |  |  |  |  |  |  |  |  |  |  |  |  |  |  |  |  |  | **↑** |  |  |  |  | **↑** |  |  |  |
| Fusobacteriaceae |  |  |  |  |  |  |  |  |  |  |  |  |  |  |  |  |  |  | **↑** |  |  |  |  |  |  |  |  |
| Geodermatophilaceae |  |  |  |  |  |  |  |  |  |  |  |  |  |  |  |  |  |  |  |  |  |  |  | **↑** |  |  |  |
| Hydrogenophilus |  |  |  |  |  |  |  |  |  |  |  |  |  |  |  |  |  |  | **↑** |  |  |  |  |  |  |  |  |
| Hyphomicrobiaceae |  |  |  |  |  |  |  |  |  |  |  |  |  |  |  |  |  |  |  |  | **↑** | **↑** |  |  |  |  |  |
| Intrasporangiaceae |  |  |  |  |  |  |  |  |  |  |  |  |  |  |  |  |  |  | **↑** |  |  | **↑** |  | **↑** |  |  |  |
| Lachnospiraceae |  |  |  |  |  |  |  |  |  |  |  |  |  |  |  |  |  |  |  | **↑** |  |  |  |  |  |  |  |
| Lactobacillaceae |  |  |  |  |  |  |  |  |  |  |  |  |  |  |  |  |  |  | **↑** |  |  | **↑** |  |  |  |  |  |
| Leptotrichiaceae |  |  |  |  |  |  |  |  |  |  |  |  |  |  |  |  |  |  |  |  | **↑** | **↑** |  |  |  |  |  |
| Leuconostocaceae |  | **↑** | **↑** |  |  |  |  |  |  |  |  |  |  |  |  |  |  |  |  |  | **↑** | **↑** |  |  |  |  |  |
| Methanobacteroaceae |  |  |  |  |  |  |  |  |  |  |  |  |  |  |  |  |  |  |  |  |  |  |  | **↑** |  |  |  |
| Methylobacteriaceae |  |  |  |  |  |  |  |  |  |  |  |  |  |  |  |  |  |  | **↑** |  |  |  |  |  |  |  |  |
| Microbacteriaceae |  |  |  |  |  |  |  |  |  |  |  |  |  |  |  |  |  |  | **↑** |  |  |  |  | **↑** |  |  |  |
| Micrococcaceae |  |  |  |  |  |  |  |  |  |  |  |  |  |  |  |  |  |  |  |  | **↑** | **↑** |  | **↑** |  |  |  |
| Mycobacteriaceae |  |  |  |  |  |  |  |  |  |  |  |  |  |  |  |  |  |  |  |  | **↑** | **↑** |  | **↑** |  |  |  |
| Neisseriaceae |  |  | **↑** |  |  |  |  |  |  |  |  |  |  |  |  |  |  |  |  |  |  |  |  |  |  |  |  |
| Nocardioidaceae |  |  |  |  |  |  |  |  |  |  |  |  |  |  |  |  |  |  |  |  |  |  |  | **↑** |  |  |  |
| Norcadiaceae |  |  |  |  |  |  |  |  |  |  |  |  |  |  |  |  |  |  |  |  |  |  |  | **↑** |  |  |  |
| Paenibacillaceae1 |  |  |  |  |  |  |  |  |  |  |  |  |  |  |  |  |  |  |  |  | **↑** | **↑** |  | **↑** |  |  |  |
| Peptostreptococcaceae |  |  |  |  |  |  |  |  |  |  |  |  |  |  |  |  |  |  |  |  | **↑** | **↑** |  |  |  |  |  |
| Phlanctomycetales |  |  |  |  |  |  |  |  |  |  |  |  |  |  |  |  |  |  |  |  |  | **↑** |  |  |  |  |  |
| Phyllobacteriaceae |  |  |  |  |  |  |  |  |  |  |  |  |  |  |  |  |  |  |  |  | **↑** |  |  |  |  |  |  |
| Planococcaceae |  |  |  |  |  |  |  |  |  |  |  |  |  |  |  |  |  |  | **↑** |  |  |  |  | **↑** |  |  |  |
| Plw_20 |  | **↓** |  |  |  |  |  |  |  |  |  |  |  |  |  |  |  |  |  |  |  |  |  |  |  |  |  |
| Porphyromonadaceae |  |  |  |  |  |  |  |  |  |  |  |  |  |  |  |  |  |  | **↑** |  |  |  |  |  |  |  |  |
| Prevotellaceae |  |  |  |  |  |  |  |  |  |  |  |  |  |  |  |  |  |  |  |  |  |  | **↑** |  | **↑** |  |  |
| Proprionibacteriaceae |  | **↓** |  |  |  |  |  |  |  |  |  |  |  |  |  |  |  |  |  |  |  |  |  | **↑** |  |  |  |
| Pseudomonadaceae |  | **↑** |  |  |  |  |  |  |  |  |  |  |  |  |  |  |  |  |  |  |  |  |  |  |  |  |  |
| Pseudonocardiaceae |  |  |  |  |  |  |  |  |  |  |  |  |  |  |  |  |  |  |  |  |  |  |  | **↑** |  |  |  |
| Rhizobiaceae |  |  |  |  |  |  |  |  |  |  |  |  |  |  |  |  |  |  | **↑** |  |  |  |  |  |  |  |  |
| Rhodobacteraceae |  |  |  |  |  |  |  |  |  |  |  |  |  |  |  |  |  |  |  |  | **↑** |  |  |  |  |  |  |
| Rhodospirillaceae |  |  |  |  |  |  |  |  |  |  |  |  |  |  |  |  |  |  |  |  | **↑** |  |  |  |  |  |  |
| Rikenellaceae |  |  |  |  |  |  |  |  |  |  |  |  |  |  |  |  |  |  |  | **↑** |  |  |  |  |  |  |  |
| Ruminococcaceae |  |  |  |  |  |  |  |  |  |  |  |  |  |  |  | **↓** | **↓** |  |  | **↑** |  |  |  |  |  |  |  |
| Sphingomonadaceae |  |  | **↑** |  |  |  |  |  |  |  |  |  |  |  |  |  |  |  | **↑** |  |  |  |  |  |  |  |  |
| Sphingobacteriaceae |  |  |  |  |  |  |  |  |  |  |  |  |  |  |  |  |  |  |  |  | **↑** | **↑** |  | **↑** |  |  |  |
| Staphylococcaceae |  |  |  |  |  |  |  |  |  |  |  |  |  |  |  |  |  |  |  |  | **↑** | **↑** |  | **↑** |  |  |  |
| Streptococcaceae |  |  |  |  |  |  |  |  |  |  |  |  |  |  |  |  |  |  | **↑** |  |  | **↑** |  |  |  |  |  |
| Streptomycetaceae |  |  |  |  |  |  |  |  |  |  |  |  |  |  |  |  |  |  | **↑** |  |  |  |  | **↑** |  |  |  |
| Trueperaceae |  |  |  |  |  |  |  |  |  |  |  |  |  |  |  |  |  |  |  |  |  |  |  | **↑** |  |  |  |
| Veillonellaceae |  |  |  |  |  |  |  |  |  |  |  |  |  |  |  |  |  |  |  |  |  |  |  |  |  |  | **↓** |
| Victivallaceae |  |  |  |  |  |  |  |  |  |  |  |  |  |  |  |  |  |  |  |  |  | **↑** |  |  |  |  |  |
| **Genus** | | | | | | | | | | | | | | | | | | | | | | | | | | | |
| *Akkermansia* |  |  |  |  |  |  |  |  |  |  |  |  |  |  |  |  |  |  |  |  |  |  |  |  |  |  | **↓** |
| *Ammonifex* |  | **↓** |  |  |  |  |  |  |  |  |  |  |  |  |  |  |  |  |  |  |  |  |  |  |  |  |  |
| *Anaerosinus* |  |  |  |  |  |  |  |  |  | **↑** |  |  |  |  |  |  |  |  |  |  |  |  |  |  |  |  |  |
| *Bacillariophyta* |  |  |  |  |  |  |  |  |  |  |  |  |  |  |  |  |  |  |  |  | **↑** | **↑** |  |  |  |  |  |
| *Bacteriodes* |  |  |  |  |  |  |  |  |  |  |  |  |  |  |  | **↓** |  | **↓** |  |  |  |  |  |  |  |  |  |
| *Bacteriodetesvadinha17_u_g* |  |  | **↓** |  |  |  |  |  |  |  |  |  |  |  |  |  |  |  |  |  |  |  |  |  |  |  |  |
| *Bacteroidales* |  |  |  |  |  |  |  |  |  |  |  |  |  |  |  | **↓** |  |  |  |  |  |  |  |  |  |  |  |
| *Barnesiellaceae* |  |  |  |  |  |  |  |  |  |  |  |  |  |  |  | **↓** | **↓** |  |  |  |  |  |  |  |  |  |  |
| *Bifidobacterium* | = |  |  |  |  |  |  |  |  | **↑** |  |  |  |  |  |  |  |  |  |  |  |  |  |  |  |  |  |
| *Blautia* |  |  |  |  |  |  |  |  |  |  |  |  |  |  |  |  |  | **↑** |  |  |  |  |  |  |  |  |  |
| *Blautia_A* |  |  |  |  |  | **↑** |  |  |  |  |  |  |  |  |  |  |  |  |  |  |  |  |  |  |  |  |  |
| *Catenibacterium* |  |  |  |  |  |  |  |  |  | **↓** |  |  |  |  |  |  |  |  |  |  |  |  |  |  |  |  |  |
| *Citrobacter* |  |  | **↑** |  |  |  |  |  |  |  |  |  |  |  |  |  |  |  |  |  |  |  |  |  |  |  |  |
| *Clostridia_u_g* |  |  | **↓** |  |  |  |  |  |  |  |  |  |  |  |  |  |  |  |  |  |  |  |  |  |  |  |  |
| *Clostridiales* |  |  |  |  |  |  |  |  |  |  |  |  |  |  |  | **↓** | **↓** |  |  |  |  |  |  |  |  |  |  |
| *Coprococcus* |  |  |  |  |  |  |  |  |  |  |  |  |  |  |  |  |  | **↑** |  |  |  |  |  |  |  |  |  |
| *Coprococcus 1* |  | **↓** |  |  |  |  |  |  |  |  |  |  |  |  |  |  |  |  |  |  |  |  |  |  |  |  |  |
| *Coprococcus_2* |  |  |  |  |  |  |  |  |  |  |  |  |  |  |  |  |  |  |  |  |  |  |  |  |  |  | **↓** |
| *Dialister* |  |  |  |  |  |  |  |  |  |  |  |  |  |  |  |  |  |  |  |  |  |  |  |  |  |  | **↓** |
| *Dorea* |  |  |  |  |  |  |  |  |  |  |  |  |  |  |  |  |  | **↑** |  |  |  |  |  |  |  |  |  |
| *Enterobacteriaceae_u_g* |  |  |  | **↑** |  |  |  |  |  |  |  |  |  |  |  |  |  |  |  |  |  |  |  |  |  |  |  |
| *Escherichia Shigella* |  |  |  |  |  |  |  |  |  |  | = | = | **↑** | **↓** | **↓** |  |  |  |  |  |  |  |  |  |  |  |  |
| *Eubacterium rectale* |  |  |  |  | **↓** |  |  |  |  |  |  |  |  |  |  |  |  |  |  |  |  |  |  |  |  |  |  |
| *Eubacterium._eligens* |  |  |  |  | **↓** |  |  |  |  |  | = | = |  |  |  |  |  |  |  |  |  |  |  |  |  |  |  |
| *Eubacterium_oxidoreducensgroup* |  |  | ↑ |  |  |  |  |  |  |  |  |  |  |  |  |  |  |  |  |  |  |  |  |  |  |  |  |
| *Faecalibacterium* |  |  |  |  |  |  |  |  |  |  | = | = | **↓** | **↑** | **↑** | **↓** | **↓** | **↓** |  |  |  |  |  |  |  |  |  |
| *Faecalibacterium prausnitzii* | **↓** | = | = | ↑ | ↓ |  |  |  |  |  |  |  |  |  |  |  |  |  |  |  |  |  |  |  |  |  |  |
| *Gp3* |  |  |  |  |  |  |  |  |  |  |  |  |  |  |  |  |  |  |  |  |  |  |  | **↑** |  |  |  |
| *Gp4* |  |  |  |  |  |  |  |  |  |  |  |  |  |  |  |  |  |  |  |  |  |  |  | **↑** |  |  |  |
| *Gp6* |  |  |  |  |  |  |  |  |  |  |  |  |  |  |  |  |  |  |  |  |  | **↑** |  | **↑** |  |  |  |
| *Kitasatospora* |  | **↓** |  |  |  |  |  |  |  |  |  |  |  |  |  |  |  |  |  |  |  |  |  |  |  |  |  |
| *Lachnospiraceae_ND3007_group* |  |  |  |  |  |  |  |  |  |  |  |  |  |  |  |  |  |  |  |  |  |  |  |  |  |  | **↓** |
| *Lachnospiraceae_uncl.* |  |  |  |  |  |  |  |  |  |  |  |  |  |  |  | **↓** | **↓** |  |  |  |  |  |  |  |  |  |  |
| *Leuconostoc* |  | **↑** |  |  |  |  |  |  |  |  |  |  |  |  |  |  |  |  |  |  |  |  |  |  |  |  |  |
| *Morganella* |  | **↑** |  |  |  |  |  |  |  |  |  |  |  |  |  |  |  |  |  |  |  |  |  |  |  |  |  |
| *Oxobacter* |  | **↓** |  |  |  |  |  |  |  |  |  |  |  |  |  |  |  |  |  |  |  |  |  |  |  |  |  |
| *Padulibacterium* |  | **↑** |  |  |  |  |  |  |  |  |  |  |  |  |  |  |  |  |  |  |  |  |  |  |  |  | **↑** |
| *Parabacteroides* |  |  |  |  |  |  |  |  |  |  |  |  |  |  |  |  |  | **↑** |  |  |  |  |  |  |  |  |  |
| *Paraprevotella* |  |  |  |  |  |  |  |  |  |  | = | = | **↑** |  |  |  |  |  |  |  |  |  |  |  |  |  |  |
| *Plw_20_u_g* |  | **↓** |  |  |  |  |  |  |  |  |  |  |  |  |  |  |  |  |  |  |  |  |  |  |  |  |  |
| *Prevotella* |  | **↑** | = | = |  |  |  |  |  |  |  |  |  |  |  | **↓** | **↑** |  |  |  |  |  |  |  |  |  |  |
| *Prevotella_9* |  |  |  |  |  |  |  |  |  |  |  |  |  |  |  |  |  |  |  |  |  |  |  |  |  |  | **↓** |
| *Proprionibacteriaceae_u_g* |  | **↓** |  |  |  |  |  |  |  |  |  |  |  |  |  |  |  |  |  |  |  |  |  |  |  |  |  |
| *Pseudomonas* |  | **↑** |  |  |  |  |  |  |  |  |  |  |  |  |  |  |  |  |  |  |  |  |  |  |  |  |  |
| *Rikenellaceae* |  |  |  |  |  |  |  |  |  |  |  |  |  |  |  | **↓** |  |  |  |  |  |  |  |  |  |  |  |
| *Rikenellaceae_RC9_gut_group* |  |  |  |  |  |  |  |  |  |  |  |  |  |  |  |  |  |  |  |  |  |  |  |  |  |  | **↓** |
| *Ruminiclostridium_5* |  |  |  |  |  |  |  |  |  |  |  |  |  |  |  |  |  |  |  |  |  |  |  |  |  |  | **↑** |
| *Ruminococcaceae_UCG* |  |  |  |  |  |  |  |  |  |  |  |  |  |  |  |  |  |  |  |  |  |  |  |  |  |  |  |
| *Ruminococcus* |  |  |  |  |  |  |  |  |  |  |  |  |  |  |  | **↓** | **↓** | **↑** |  |  |  |  |  |  |  |  |  |
| *Shingomonas* |  |  | **↑** |  |  |  |  |  |  |  |  |  |  |  |  |  |  |  |  |  |  |  |  |  |  |  |  |
| *Streptophyta* |  |  |  |  |  |  |  |  |  |  |  |  |  |  |  |  |  |  | **↑** |  |  | **↑** |  | **↑** |  |  |  |
| *Sutterella* |  |  |  |  |  |  |  |  |  |  |  |  |  |  |  |  |  | **↓** |  |  |  |  |  |  |  |  |  |
| *Weissella* |  | **↑** | **↑** |  |  |  |  |  |  |  |  |  |  |  |  |  |  |  |  |  |  |  |  |  |  |  |  |
| **Species** | | | | | | | | | | | | | | | | | | | | | | | | | | | |
| *Anaeromassilibacillus sp. An250* |  |  |  |  |  |  |  | **↓** |  |  |  |  |  |  |  |  |  |  |  |  |  |  |  |  |  |  |  |
| *Alistipes finegoldii* |  |  |  |  |  | **↑** |  |  |  |  |  |  |  |  |  |  |  |  |  |  |  |  |  |  |  |  |  |
| *Alistipes shahii* |  |  |  |  |  |  | **↑** | **↑** |  |  |  |  |  |  |  |  |  |  |  |  |  |  |  |  |  |  |  |
| *Alistipes sp. CAG:268* |  |  |  |  |  |  | **↓** |  | **↓** |  |  |  |  |  |  |  |  |  |  |  |  |  |  |  |  |  |  |
| *An114 sp002161055* |  |  |  |  |  | **↓** |  |  |  |  |  |  |  |  |  |  |  |  |  |  |  |  |  |  |  |  |  |
| *Anaerosinus glycerin* |  |  |  |  |  |  |  |  |  | **↑** |  |  |  |  |  |  |  |  |  |  |  |  |  |  |  |  |  |
| *Bacteroides dorei* |  |  |  |  |  |  | **↑** |  | **↑** |  |  |  |  |  |  |  |  |  |  |  |  |  |  |  |  |  |  |
| *Bacteroides dorei CAG:222* |  |  |  |  |  |  | **↑** | **↑** |  |  |  |  |  |  |  |  |  |  |  |  |  |  |  |  |  |  |  |
| *Bacteroides ovatus* |  |  |  |  |  |  |  |  | **↑** |  |  |  |  |  |  |  |  |  |  |  |  |  |  |  |  |  |  |
| *Bacteroides stercoris* |  |  |  |  |  |  | **↓** |  | **↓** |  |  |  |  |  |  |  |  |  |  |  |  |  |  |  |  |  |  |
| *Bacteriodes vulgatus* |  |  |  |  | **↑** |  | **↑** |  | **↑** |  |  |  |  |  |  |  |  |  |  |  |  |  |  |  |  |  |  |
| *Bacteroides xylanisolvens* |  |  |  |  |  |  | **↓** |  | **↓** |  |  |  |  |  |  |  |  |  |  |  |  |  |  |  |  |  |  |
| *Bifidobacterium adolescentis* | **↓** |  |  |  |  |  |  |  |  |  |  |  |  |  |  |  |  |  |  |  |  |  |  |  |  |  |  |
| *Bifidobacterium animalis* |  |  |  |  |  | **↓** |  |  |  |  |  |  |  |  |  |  |  |  |  |  |  |  |  |  |  |  |  |
| *Bifidobacterium dentium* | **↓** |  |  |  |  |  |  |  |  |  |  |  |  |  |  |  |  |  |  |  |  |  |  |  |  |  |  |
| *Bifidobacterium longum* | **↑** |  |  |  |  |  |  |  |  |  |  |  |  |  |  |  |  |  |  |  |  |  |  |  |  |  |  |
| *Bilophila sp. 4_1_30* |  |  |  |  |  |  | **↑** |  |  |  |  |  |  |  |  |  |  |  |  |  |  |  |  |  |  |  |  |
| *Blautia sp. CAG:52* |  |  |  |  |  |  |  | **↓** |  |  |  |  |  |  |  |  |  |  |  |  |  |  |  |  |  |  |  |
| *Blautia_A hydrogenotrophica* |  |  |  |  |  | **↓** |  |  |  |  |  |  |  |  |  |  |  |  |  |  |  |  |  |  |  |  |  |
| *Blautia_A sp000285855_ASV1* |  |  |  |  |  | **↓** |  |  |  |  |  |  |  |  |  |  |  |  |  |  |  |  |  |  |  |  |  |
| *Blautia_A sp900066505* |  |  |  |  |  | **↑** |  |  |  |  |  |  |  |  |  |  |  |  |  |  |  |  |  |  |  |  |  |
| *Blautia obeum* |  |  |  |  |  |  | **↑** | **↑** |  |  |  |  |  |  |  |  |  |  |  |  |  |  |  |  |  |  |  |
| *Blautia wexlerae* |  |  |  |  |  |  |  |  | **↓** |  |  |  |  |  |  |  |  |  |  |  |  |  |  |  |  |  |  |
| *CAG-81 sp900066055* |  |  |  |  |  | **↑** |  |  |  |  |  |  |  |  |  |  |  |  |  |  |  |  |  |  |  |  |  |
| *Clostridium clostridioforme* |  |  |  |  |  |  |  | **↑** |  |  |  |  |  |  |  |  |  |  |  |  |  |  |  |  |  |  |  |
| *Clostridium_M bolteae* |  |  |  |  |  | **↑** |  |  |  |  |  |  |  |  |  |  |  |  |  |  |  |  |  |  |  |  |  |
| *Clostridium_M bolteae_ASV1* |  |  |  |  |  | **↑** |  |  |  |  |  |  |  |  |  |  |  |  |  |  |  |  |  |  |  |  |  |
| *Collinsella aerofaciens* |  |  |  |  |  |  | **↓** | **↓** |  |  |  |  |  |  |  |  |  |  |  |  |  |  |  |  |  |  |  |
| *Collinsella intestinalis* |  |  |  |  |  |  | **↓** | **↓** |  |  |  |  |  |  |  |  |  |  |  |  |  |  |  |  |  |  |  |
| *Collinsella stercoris* |  |  |  |  |  |  | **↓** | **↓** |  |  |  |  |  |  |  |  |  |  |  |  |  |  |  |  |  |  |  |
| *Coprococcus comes* |  |  |  |  |  |  |  |  | **↑** |  |  |  |  |  |  |  |  |  |  |  |  |  |  |  |  |  |  |
| *Enterorhabdus caecimuris* |  |  |  |  |  |  |  | **↑** |  |  |  |  |  |  |  |  |  |  |  |  |  |  |  |  |  |  |  |
| *Erysipelatoclostridium ramosum* |  |  |  |  |  |  |  | **↑** |  |  |  |  |  |  |  |  |  |  |  |  |  |  |  |  |  |  |  |
| *Eubacterium eligens* |  |  |  |  |  |  | **↑** | **↑** | **↑** |  |  |  |  |  |  |  |  |  |  |  |  |  |  |  |  |  |  |
| *Eubacterium sp. CAG:38* |  |  |  |  |  |  | **↓** | **↓** |  |  |  |  |  |  |  |  |  |  |  |  |  |  |  |  |  |  |  |
| *Faecalibacterium prausnitzii* |  |  |  |  |  |  | **↑** |  | **↑** |  |  |  |  |  |  |  |  |  |  |  |  |  |  |  |  |  |  |
| *Firmicutes bacterium CAG:65* |  |  |  |  |  |  | **↑** | **↑** | **↓** |  |  |  |  |  |  |  |  |  |  |  |  |  |  |  |  |  |  |
| *Flavonifractor plautii* |  |  |  |  |  | **↑** | **↓** |  |  |  |  |  |  |  |  |  |  |  |  |  |  |  |  |  |  |  |  |
| *Fusicatenibacter saccharivorans* |  |  |  |  |  |  |  |  | **↑** |  |  |  |  |  |  |  |  |  |  |  |  |  |  |  |  |  |  |
| *GCA-900066135 sp900066135* |  |  |  |  |  | **↓** |  |  |  |  |  |  |  |  |  |  |  |  |  |  |  |  |  |  |  |  |  |
| *Klebsiella pneumoniae* |  |  |  |  |  |  |  | **↓** |  |  |  |  |  |  |  |  |  |  |  |  |  |  |  |  |  |  |  |
| *Lactococcus lactis* |  |  |  |  |  | **↑** |  |  |  |  |  |  |  |  |  |  |  |  |  |  |  |  |  |  |  |  |  |
| *Massilioclostridium methylpentosum* |  |  |  |  |  | **↓** |  |  |  |  |  |  |  |  |  |  |  |  |  |  |  |  |  |  |  |  |  |
| *Parabacteroides distasonis* |  |  |  |  |  |  | **↓** | **↓** | **↓** |  |  |  |  |  |  |  |  |  |  |  |  |  |  |  |  |  |  |
| *Parabacteroides merdae* |  |  |  |  |  |  |  |  | **↓** |  |  |  |  |  |  |  |  |  |  |  |  |  |  |  |  |  |  |
| *Prevotella copri_A* |  |  |  |  |  | **↓** |  |  |  |  |  |  |  |  |  |  |  |  |  |  |  |  |  |  |  |  |  |
| *Prevotella sp. CAG:386* |  |  |  |  |  |  |  |  | **↓** |  |  |  |  |  |  |  |  |  |  |  |  |  |  |  |  |  |  |
| *Roseburia hominis* |  |  |  |  |  |  | **↑** | **↑** |  |  |  |  |  |  |  |  |  |  |  |  |  |  |  |  |  |  |  |
| *Roseburia inulinivorans* |  |  |  |  |  |  |  | **↑** |  |  |  |  |  |  |  |  |  |  |  |  |  |  |  |  |  |  |  |
| *Roseburia sp. CAG:45* |  |  |  |  |  |  |  |  | **↓** |  |  |  |  |  |  |  |  |  |  |  |  |  |  |  |  |  |  |
| *Ruminococcus torques* |  |  |  |  |  |  |  | **↓** |  |  |  |  |  |  |  |  |  |  |  |  |  |  |  |  |  |  |  |
| *Ruminococcus gnavus* |  |  |  |  |  |  |  |  | **↓** |  |  |  |  |  |  |  |  |  |  |  |  |  |  |  |  |  |  |
| *Ruminococcus_E bromii* |  |  |  |  |  | **↑** |  |  |  |  |  |  |  |  |  |  |  |  |  |  |  |  |  |  |  |  |  |
| *Streptococcus agalactiae* |  |  |  |  | **↑** |  |  |  |  |  |  |  |  |  |  |  |  |  |  |  |  |  |  |  |  |  |  |
| *Streptococcus australis* |  |  |  |  |  | **↓** |  |  |  |  |  |  |  |  |  |  |  |  |  |  |  |  |  |  |  |  |  |
| *Terrisporobacter othiniensis* |  |  |  |  |  | **↑** |  |  |  |  |  |  |  |  |  |  |  |  |  |  |  |  |  |  |  |  |  |
| *Veillonella dispar_ASV1* |  |  |  |  |  | **↓** |  |  |  |  |  |  |  |  |  |  |  |  |  |  |  |  |  |  |  |  |  |
| *Veillonella parvula* |  |  |  |  |  |  | **↓** | **↓** |  |  |  |  |  |  |  |  |  |  |  |  |  |  |  |  |  |  |  |
| *Veillonella tobetsuensis* |  |  |  |  |  | **↓** |  |  |  |  |  |  |  |  |  |  |  |  |  |  |  |  |  |  |  |  |  |
| **p-value adjustment** | BH | Not reported | | | BC | Not reported | BH | | | Not reported | BH | | | | | BH | | | BH | | | | | | | BH | BH |

**NOTE:** A significant decrease (red box and down arrow), a significant increase (green box and up arrow) or no significant difference (yellow and equal sign) in abundance were reported.

**^Ⴕ^** Denotes were linear discriminate analysis effect size (LEfSe) analysis was used.

CD, Crohn’s disease; UC, ulcerative colitis; FODMAP, fermentable oligosaccharides, disaccharides, monosaccharides, and polyols; GOS, galacto-oligosaccharide; LFD, low-fat diet; iSAD, improved Standard American Diet; SOC, standard-of-care; DD, diversified diet; NDD, non-diversified diet; SCD, Specific Carbohydrate Diet; GFD, gluten-free diet; BH, Benjamini-Hochberg; BC, Bonferroni correction.

**Supplementary Table S6.** Taxonomy of Relevant Bacteria Identified in the Included Studies

| **Phylum** | Acidobacteria | Actinobacteria | Bacteriodetes | Chlamydiota | Cyanobacteria | Deinococcota | Deferribacterota | Fibrobacterota | Firmicutes | Fusoacteriota | Proteobacteria | Verrucomicrobia | Lentisphaerota |
| --- | --- | --- | --- | --- | --- | --- | --- | --- | --- | --- | --- | --- | --- |
| **Class** | Acidobacteria  Acidobacteria_Gp3  Acidobacteria_Gp6  Acidobacteria_Gp4 | Actinobacteria  Coriobacteriaceae | Bacteroidia  Cytophagia  Erysipelotrichia  Flavobacteriia | Chlamydiales  Chlamydiia | Chloroplast  Cyanobacteria | Deinococci | Deferribacteres | Fibrobacteria | Bacilli  Clostridia | Fusobacteriia |  | Verrucomicrobiae |  |
| **Order** |  | Acidimicrobiales  Actinomycetales  Bifidobacteriales  Coriobacteriales | Bacteriodales  Cytophagales  Flavobacteriales  Sphingobacteriales  Sphingomonadales | Chlamydiales | Chloroplast | Deinococcales  Deinococci | Deferribacterales |  | Bacillales  Clostriadiales  Clostridia_u_o  Erysipelotrichales  Lactobacillales  Selenomonadales | Fusobacteriales | Caulobacterales  Methylococcales  Neisseriales  Pseudomonadales  Rhizobiales  Rhodobacterales  Rhodospirillales | Verrucomicrobiales | Victivallales |
| **Family** |  | Acidimicrobiaceae  Actinomycetaceae  Atopobiaceae  Bifidobacteriaceae  Brevibacteriaceae  Cellulomonadaceae  Coriobacteriaceae  Corynebacteriaceae  Dermacoccaceae  Dietziaceae  Geodermatophilaceae  Intrasporangiaceae  Microbacteriaceae  Micrococcaceae  Mycobacteriaceae  Nocardioidaceae  Norcadiaceae  Proprionibacteriaceae  Pseudonocardiaceae  Streptomycetaceae | Bacteroidaceae  Chitinophagaceae  Cryomorphaceae  Cytophagaceae  Flavobacteriaceae  Porphyromonadaceae  Prevotellaceae  Rikenellaceae  Shingomonadaeceae  Sphingobacteriaceae |  | Chloroplast | Deinococcaceae  Trueperaceae | Deferribacteraceae |  | Aerococcaceae  Bacillaceae1  Bacillales_IncertaeSedisXI  Bacillales_IncertaeSedisXII  Barnesiellaceae  Carnobacteriaceae  Clostriadiales_IncertaeSedisXI  Clostriadiales_IncertaeSedisXIII  Clostridiaceae1  Clostridia_u_f  Defluvitaleaceae  Enterococcaceae  Erysipelotrichaceae  Eubacteriaceae  Lachnospiraceae  Lactobacillaceae  Leuconostocaceae  Paenibacillaceae1  Peptostreptococcaceae  Planococcaceae  Ruminococcaceae  Staphylococcaceae  Streptococcaceae  Veillonellaceae | Fusobacteriaceae  Leptotrichiaceae | Bradyrhizobiaceae  Brucellaceae  Caulobacteraceae  Dermabacteraceae  Enterobacteriaceae  Fibrobacteraceae  Hyphomicrobiaceae  Methylobacteriaceae  Neisseriaceae  Plw_20  Phyllobacteriaceae  Pseudomonadaceae  Rhizobiaceae  Rhodobacteraceae  Rhodospirillaceae  Sphingomonadaceae | Akkermansiaceae | Victivallaceae |
| **Genus** | *Gp3*  *Gp4*  *Gp6* | *Bifidobacterium*  *Kitasatospora*  *Leuconostoc*  *Proprionibacteriaceae_u_g* | *Bacteriodes*  *Bacteroidales*  *Bacteriodetesvadinha17_u_g*  *Barnesiellaceae*  *Parabacteroides*  *Paraprevotella*  *Prevotella*  *Prevotella 9*  *Rikenellaceae*  *Rikenellaceae_RC9_gut_ group* |  |  |  |  |  | *Ammonifex*  *Anaerosinus*  *Blautia*  *Blautia A*  *Catenibacterium*  *Clostridiales*  *Clostridia_u_g*  *Coprococcus*  *Coprococcus 1*  *Coprococcus 2*  *Dialister*  *Dorea*  *Eubacterium rectale*  *Eubacterium eligens*  *Eubacterium_oxidoreducens group*  *Faecalibacterium*  *Faecalibacterium prausnitzii*  *Lachnospiraceae ND3007 group*  *Lachnospiraceae_uncl.*  *Leuconostoc*  *Oxobacter*  *Ruminiclostridium_5*  *Ruminococcaceae_UCG*  *Ruminococcus*  *Weissella* |  | *Citrobacter*  *Escherichia Shigella*  *Enterobacteriaceae_u_g*  *Morganella*  *Plw_20_u_g*  *Pseudomonas*  *Shingomonas*  *Sutterella* | *Akkermansia* |  |
| **Species** |  | *Bifidobacterium adolescentis*  *Bifidobacterium animalis*  *Bifidobacterium dentium*  *Bifidobacterium longum*  *Collinsella aerofaciens*  *Collinsella intestinalis*  *Collinsella stercoris*  *Enterorhabdus caecimuris* | *Alistipes finegoldii*  *Alistipes shahii*  *Alistipes sp. CAG:268*  *Bacteroides dorei*  *Bacteroides dorei CAG:222*  *Bacteroides ovatus*  *Bacteroides stercoris*  *Bacteriodes vulgatus*  *Bacteroides xylanisolvens*  *Parabacteroides distasonis*  *Parabacteroides merdae*  *Prevotella copri_A*  *Prevotella sp. CAG:386* |  |  |  |  |  | *Anaeromassilibacillus sp. An250*  *Blautia sp. CAG:52*  *Blautia_A hydrogenotrophica*  *Blautia_A sp000285855_ASV1*  *Blautia_A sp900066505*  *Blautia obeum*  *Blautia wexlerae*  *CAG-81 sp900066055*  *Clostridium clostridioforme*  *Clostridium_M bolteae*  *Clostridium_M bolteae_ASV1*  *Coprococcus comes*  *Erysipelatoclostridium ramosum*  *Eubacterium eligens*  *Eubacterium sp. CAG:38*  *Faecalibacterium prausnitzii*  *Firmicutes bacterium CAG:65*  *Flavonifractor plautii*  *Fusicatenibacter saccharivorans*  *GCA-900066135 sp900066135*  *Lactococcus lactis*  *Massilioclostridium methylpentosum*  *Roseburia hominis*  *Roseburia inulinivorans*  *Roseburia sp. CAG:45*  *Ruminococcus torques*  *Ruminococcus gnavus*  *Ruminococcus_E bromii*  *Streptococcus agalactiae*  *Streptococcus australis*  *Terrisporobacter othiniensis*  *Veillonella dispar_ASV1*  *Veillonella parvula*  *Veillonella tobetsuensis* |  | *Klebsiella pneumoniae* |  |  |

**Abbreviations used in this paper:**

| **AMeD** | Alternate Mediterranean Diet Score |
| --- | --- |
| **ASA-24** | Automated Self-Administered 24-hour dietary recall |
| **BASFI** | Bath Ankylosing Spondylitis Functional Index |
| **BC** | Bonferroni correction |
| **BH** | Benjamini-Hochberg |
| **CD** | Crohn's disease |
| **CHD** | Canadian Habitual Diet |
| **CNHS2010-F** | 2010 Chinese Residents of Nutrition and Health Status Monitoring  Semi-Quantitative Food Questionnaire |
| **CRP** | C-reactive protein |
| **DD** | diversified diet |
| **EEN** | exclusive enteral nutrition |
| **FC** | faecal calprotectin |
| **FFQ** | food frequency questionnaire |
| **FODMAP** | fermentable oligosaccharides, disaccharides, monosaccharides, and polyols |
| **FT** | faecal transplant |
| **GFD** | gluten-free diet |
| **GI** | gastrointestinal |
| **GM-CSF** | Granulocyte-macrophage colony-stimulating factor |
| **GOS** | galacto-oligosaccharide |
| **GSRS** | GI symptom rating scale |
| **HBI** | Harvey-Bradshaw Index |
| **HC** | healthy control |
| **HE-2015** | Healthy Eating Index 2015 |
| **hsCRP** | high sensitivity C-reactive protein |
| **IBD** | inflammatory bowel disease |
| **IBD-AID** | Inflammatory Bowel Disease-Anti-Inflammatory Diet |
| **IBS-SSS** | Irritable Bowel Syndrome Severity System Scores |
| **IHMS** | International Human Microbiome Standards |
| **SOP** | Standard Operating Procedure |
| **IL1β** | interleukin 1 beta |
| **INF-γ** | interferon gamma |
| **iSAD** | improved Standard American Diet |
| **LFD** | low-fat diet |
| **MARS** | Medication Adherence Rating Scale |
| **MDS** | Mediterranean Diet Score |
| **MDSS** | Mediterranean Diet Serving Score |
| **MGS** | metagenomic shotgun sequencing |
| **MUFA** | monounsaturated fatty acid |
| **NCBI** | National Centre for Biotechnology Information |
| **NDD** | non-diversified diet |
| **OTUs** | operational taxonomic units |
| **PCoA** | principal coordinate analysis |
| **PERMANOVA** | permutational multivariate analysis of variance |
| **PUFA** | polyunsaturated fatty acid |
| **PMS** | Partial Mayo Score |
| **PRISMA** | Preferred Reporting Items for Systematic reviews and Meta-Analyses |
| **PROMIS** | Patient-Reported Outcomes Management Information System |
| **PSP** | Pre-analytical Sample Processing |
| **QIIME** | Quantitative Insights Into Microbial Ecology |
| **QoL** | quality of life |
| **RAPID-3** | Routine Assessment of Patient Index Data 3 |
| **RCT** | randomised controlled trial |
| **SAA** | serum amyloid A |
| **SCCAI** | simple clinical colitis activity index |
| **SCD** | Specific Carbohydrate Diet |
| **sCDAI** | short Crohn’s Disease Activity Index |
| **SF-36** | Short Form-36 Health Survey |
| **SGB** | species-level genome bins |
| **sIgA** | secretory immunoglobulin A |
| **SIBDQ** | Short Inflammatory Bowel Disease |
| **SOC** | standard-of-care |
| **THSTI** | Translational Health Sciences and Technology Institute |
| **TNF-α** | tumour necrosis factor alpha |
| **UC** | ulcerative colitis |
| **UCED** | Ulcerative Colitis Exclusion Diet |
| **WD** | Western diet |
