# Supplementary material for: The Impact of Dietary Interventions on the Microbiota in Inflammatory Bowel Disease: A Systematic Review
Source: J Crohns Colitis. 2023 Dec 15;18(6):920–42. doi: 10.1093/ecco-jcc/jjad204 (PMC11147801; doi:10.1093/ecco-jcc/jjad204)
Supplement: jjad204_suppl_Supplementary_Table_S7 [file jjad204_suppl_supplementary_table_s7.docx]

**Table S7.** Summary of Findings: Changes in Microbial Diversity Indices in IBD Patients with Diet

| **Study** | Cox *et al*., 2020^39^ | | Fritsch *et al*., 2021^40^ | | Lewis *et al*., 2021^41^ | | Haskey *et al*., 2023^49^ | Strauss *et al.,* 2023^50^ | Olendzki *et at*. 2022^51^ | Sahu *et al*., 2021^42^ | | Zhang *et al*., 2020^44^ | | | | Shabat *et al*., 2021^43^ | Schreiner *et al*., 2019^46^ | | Weng *et al*., 2020^48^ | | | Teofani *et al*., 2022^47^ | Berbisá *et al*., 2022^45^ |
| --- | --- | --- | --- | --- | --- | --- | --- | --- | --- | --- | --- | --- | --- | --- | --- | --- | --- | --- | --- | --- | --- | --- | --- |
| **Diet intervention and/or comparator** | Low FODMAP diet  (CD/UC, *n* = 21)  vs.  Sham diet  (CD/UC, *n* = 22)  [End-of-trial, wk 8] | LFD  (UC, *n* = 17) [Baseline] | iSAD  (UC, *n* = 17)  [Baseline] | LFD  (UC, *n* = 17)  vs.  iSAD  (*n* = 17) | SCD  (CD, *n* = 99)  vs.  MD  (CD, *n* = 92)  [Baseline] | SCD  (CD, *n* = 99)  vs.  MD  (CD, *n* = 92)  [wk 6] | MD (UC, *n* = 15 ) vs. CHD (UC, *n* = 13) [end-of-trial, wk 12] | MD (UC, *n* = 22) vs. habitual diet (baseline) (UC, *n* = 18) | IBD-AID (CD/UC, *n* = 22) vs. Habitual (baseline) (CD/UC, *n* = 22) | SOC  (UC, *n* = 13)  vs.  EEN  (UC, *n* = 14)  [Baseline, Day 1] | SOC  (UC, *n* = 13)  Vs.  EEN  (UC, *n* = 14)  [End of trial, Day 7] | DD  (CD, *n* = 25)  vs.  NDD  (CD, *n* = 15)  [Baseline] | DD  (CD, *n* = 25)  vs.  NDD  (CD, *n* = 15)  [End of trial, wk 12] | DD  (CD, *n* = 25)  [Baseline vs. end of trial, wk 12] | NDD  (CD, *n* = 15)  [Baseline vs. end of trial, wk 12] | Donors  (HC, *n* = 7)  [pre-conditioning vs. post-conditioning (wk 2)] | VD (CD, *n* = 2)  vs regular diet (CD, *n* = 41) | GFD vs. (UC, *n* = 6)  vs.  regular diet (UC, n =25) | Habitual diet (CD, *n* = 58) vs.  (HC, *n* = 24) | Habitual diet (CD, *n* = 58) vs. (CD, *n* = 41) [stool vs. biopsy] | Habitual diet (UC, *n* = 31) vs. (UC, *n* = 25) [stool vs. biopsy] | Habitual diet  (CD, *n* = 52; UC, *n* = 58) vs.  (HC, *n* = 42) | Habitual diet  (UC, *n* = 41)  vs.  (HC, *n* = 144) |
| **Alpha diversity** | | | | | | | | | | | | | | | | | | | | | | | |
| **Richness** | NS  p = 0.599 |  |  |  | NS  p = 0.88 | NS  p = 0.35 | NS  p = 0.68 |  |  |  |  | NS  p ≥ 0.35 | NS |  |  | NS |  |  |  |  |  |  | NS |
| **Observed species** |  |  |  |  |  |  |  | NS |  |  |  |  |  |  |  |  |  |  |  |  |  |  |  |
| **Microbial gene richness** | NS  p = 0.281 |  |  |  |  |  |  |  |  |  |  |  |  |  |  |  |  |  |  |  |  |  |  |
| **Shannon** | NS  p = 0.228 |  |  |  | NS  p = 0.90 | NS  p = 0.26 | NS  p = 0.13 | NS | NS | NS | NS | NS  p ≥ 0.35 | NS |  |  | NS | NS  p = 0.07 | NS  p = 0.05 | **p < 0.05** | NS | NS | **p = 3.650 × 10^−9^** | NS  p = 0.99 |
| **Simpson** | NS  p = 0.461 |  |  |  |  |  |  | NS |  |  |  |  |  |  |  |  | NS | NS |  |  |  | **p = 1.617 × 10^−7^** |  |
| **Faith’s phylogenetic diversity** |  | NS  p = 0.13 | NS  p = 0.15 | NS  p = 0.78 |  |  | NS  p = 0.68 |  |  | NS | NS |  |  |  |  |  |  |  |  |  |  |  |  |
| **Pielou’s evenness** |  |  |  |  |  |  |  |  |  | NS | NS |  |  |  |  |  |  |  |  |  |  |  |  |
| **Chao1** |  |  |  |  |  |  |  |  |  |  |  |  |  |  |  |  |  |  |  |  |  | **p = 4.401 × 10^−9^** |  |
| **Observed OTUs** |  |  |  |  |  |  |  |  |  |  |  |  |  |  |  |  | NS | NS |  |  |  |  |  |
| **Beta-diversity** | | | | | | | | | | | | | | | | | | | | | | | |
| **Bray-Curtis**  **Mann-Whitney** | P = 0.585 |  |  |  |  |  |  |  |  |  |  |  |  |  |  |  |  |  |  |  |  |  |  |
| **Bray-Curtis PERMANOVA** |  | NS  p = 0.05 | NS  p = 0.40 | NS  p = 0.64 | NS  p = 0.94 | NS  p= 0.26 |  | NS | NS |  |  |  |  |  |  |  | **p = 0.012** | **p < 0.001** |  |  |  | **p < 0.0001** | NS |
| **Aitchison PERMANOVA** |  |  |  |  |  |  |  |  |  | NS  p = 0.53 | NS  p = 0.06 |  |  |  |  |  |  |  |  |  |  |  |  |
| **Manhattan and Gower PERMANOVA** |  |  |  |  |  |  |  |  |  |  |  | **p = 0.003** | NS  p = 0.43 | NS  p = 0.99 | NS  p = 0.20 |  |  |  |  |  |  |  |  |
| **Weighted Unifrac**  **PERMANOVA** |  |  |  |  |  |  |  |  |  |  |  |  |  |  |  |  |  |  |  |  |  | **p < 0.0001** |  |
| **Unweighted Unifrac**  **PERMANOVA** |  |  |  |  |  |  |  |  |  |  |  |  |  |  |  |  |  |  |  |  |  | **p < 0.0001** |  |
| **Canberra**  **PERMANOVA** |  |  |  |  |  |  |  |  |  |  |  |  |  |  |  |  |  |  |  |  |  | **p < 0.0001** |  |
| **Bray-Curtis**  **PCoA** |  |  |  |  |  |  |  |  |  |  |  |  |  |  |  |  |  |  |  |  |  |  | NS |
| **Unweighted Unifrac**  **PCoA** |  |  |  |  |  |  |  |  |  |  |  |  |  |  |  |  |  |  | NS | NS |  |  |  |

**NOTE:** Where available, p values are reported in the table. Bold text denotes statistically significant p values (p < 0.05). Blank cell = not assessed. NS = not significant.

CD, Crohn’s disease; UC, ulcerative colitis; FODMAP, fermentable oligosaccharides, disaccharides, monosaccharides, and polyols; LFD, low-fat diet; iSAD, improved Standard American Diet; SOC, standard-of-care; DD, diversified diet; NDD, non-diversified diet; SCD, Specific Carbohydrate Diet; IBD-AID, Inflammatory Bowel Disease Anti-inflammatory Diet; CHD, Canadian Habitual Diet; MD, Mediterranean diet; GFD, gluten-free diet; OTUs, operational taxonomic units; PERMANOVA, permutational multivariate analysis of variance; PCoA, principal coordinate analysis
